# In oxygen-deprived tumor cells ERp57 provides radioprotection and ensures proliferation via c-Myc, PLK1 and the AKT pathway

**DOI:** 10.1038/s41598-021-86658-5

**Published:** 2021-03-30

**Authors:** Tobias Ocklenburg, Fabian Neumann, Alexandra Wolf, Julia Vogel, Kirsten Göpelt, Melanie Baumann, Jennifer Baumann, Philip Kranz, Eric Metzen, Ulf Brockmeier

**Affiliations:** 1 Institut Für Physiologie, Universität Duisburg-Essen, Duisburg, Germany; 2 Department of Neurology, University Hospital Essen, Essen, Germany

**Keywords:** Biochemistry, Cancer, Cell biology, Molecular biology

## Abstract

The disulfide isomerase ERp57, originally found in the endoplasmic reticulum, is located in multiple cellular compartments, participates in diverse cell functions and interacts with a huge network of binding partners. It was recently suggested as an attractive new target for cancer therapy due to its critical role in tumor cell proliferation. Since a major bottleneck in cancer treatment is the occurrence of hypoxic areas in solid tumors, the role of ERp57 in cell growth was tested under oxygen depletion in the colorectal cancer cell line HCT116. We observed a severe growth inhibition when ERp57 was knocked down in hypoxia (1% O_2_) as a consequence of downregulated c-Myc, PLK1, PDPK1 (PDK1) and AKT (PKB). Further, irradiation experiments revealed also a radiosensitizing effect of ERp57 depletion under oxygen deprivation. Compared to ERp57, we do not favour PDPK1 as a suitable pharmaceutical target as its efficient knockdown/chemical inhibition did not show an inhibitory effect on proliferation.

## Introduction

Despite advances in surgical quality, chemotherapies and irradiation, colorectal cancer (CRC) is still a predominant cancer in western countries due to marginal improvements in long-term survival rates^1,2^. A typical characteristic of solid tumors are hypoxic regions that represent a negative prognostic factor for a patients outcome and are therefore of high clinical relevance. Many promising chemotherapeutics failed when exposed to a hypoxic environment^3^. Involved in chemoresistance are the hypoxia-inducible factors (HIFs) 1–3, transcription factors that are key mediators for the cellular response to hypoxia. Predominantly HIF1 and HIF2 regulate the expression of numerous genes that drive adaptation and progression of tumor cells^4^. Further, oxygen deprived tumor areas show decreased generation of reactive oxygen species during radiotherapy that causes radioresistance, recurrence and metastasis^5,6^.

To explore new targets for cancer treatment, the endoplasmic reticulum (ER) is a suitable cellular place to look for, since a general feature of cancer growth is the abnormal need for high protein levels. Exclusively in the ER, secretory proteins are synthesized, get folded and transported to their final destination in the cell membrane or outside the cell. This whole process underlies a complex quality control and is prone to ER stress which can easily lead to cell death if the ER homeostasis is irreversibly disturbed.

Originally, the thiol-oxidoreductase ERp57 (aka PDIA3, GRP58) was found as an ER-luminal protein that participates in the folding and quality control of glycoproteins, in particular in the assembly of the major histocompatibility complex class I^7^. It belongs to the family of protein disulfide isomerases (PDIs) and beside its role as a chaperone, it acts as a catalyst for disulfide bond formation in the rather oxidizing environment of the ER. Like its close homologue PDI, the ERp57 protein consists of four domains a, b, b' and a' that form an U shape structure. This arrangement brings the a and a’ domains with its thioredoxin-like active sites in close proximity to each other to facilitate the redox reactions with its client proteins. Although catalytically inactive, the b and b’ domains are mandatory for its chaperoning function^8^.

We were recently able to demonstrate in the CRC cell line HCT116 under normoxic conditions, that depletion of ERp57 alone activated exclusively the PERK branch of the Unfolded Protein Response (UPR)^9^. The UPR is an ER signaling pathway that is critical for cell survival and is initiated under stressful conditions to restore ER homeostasis^10^. As we found out later, the activation of the PERK pathway was not due to ER stress caused by an overload of misfolded proteins, but rather due to ERp57`s role as an ER redox sensor that keeps PDI in a reduced state to maintain PERK in an inactive form^11^. Further, we recognized a p53-independent impaired cancer cell growth after ERp57 deficiency. Since a proportion of ERp57 was also detected outside the ER in the cytosol^9,12,13^, a direct participation of ERp57 in cytoplasmic signal transductions was conceivable and could explain this drastic impact on cell proliferation. Although we and others noticed a positive effect of ERp57 on mTOR-complex1 phosphorylation status^12^, an overall picture of the interplay between ERp57 and growth-related pathways is still missing.

In consequence, here we intended to clarify the role of ERp57 for hypoxic cancer cell growth in combination with irradiation and tested it exemplarily in the CRC cell line HCT116. By the use of a genetically controlled ERp57 knockdown (KD) we were able to detect substantial changes in proliferation signal cascades that underline the critical part of ERp57 for tumor formation under normoxic and hypoxic condition.

## Results

### Deprivation of ERp57 induces moderate apoptosis under hypoxic conditions

**Figure 1:**
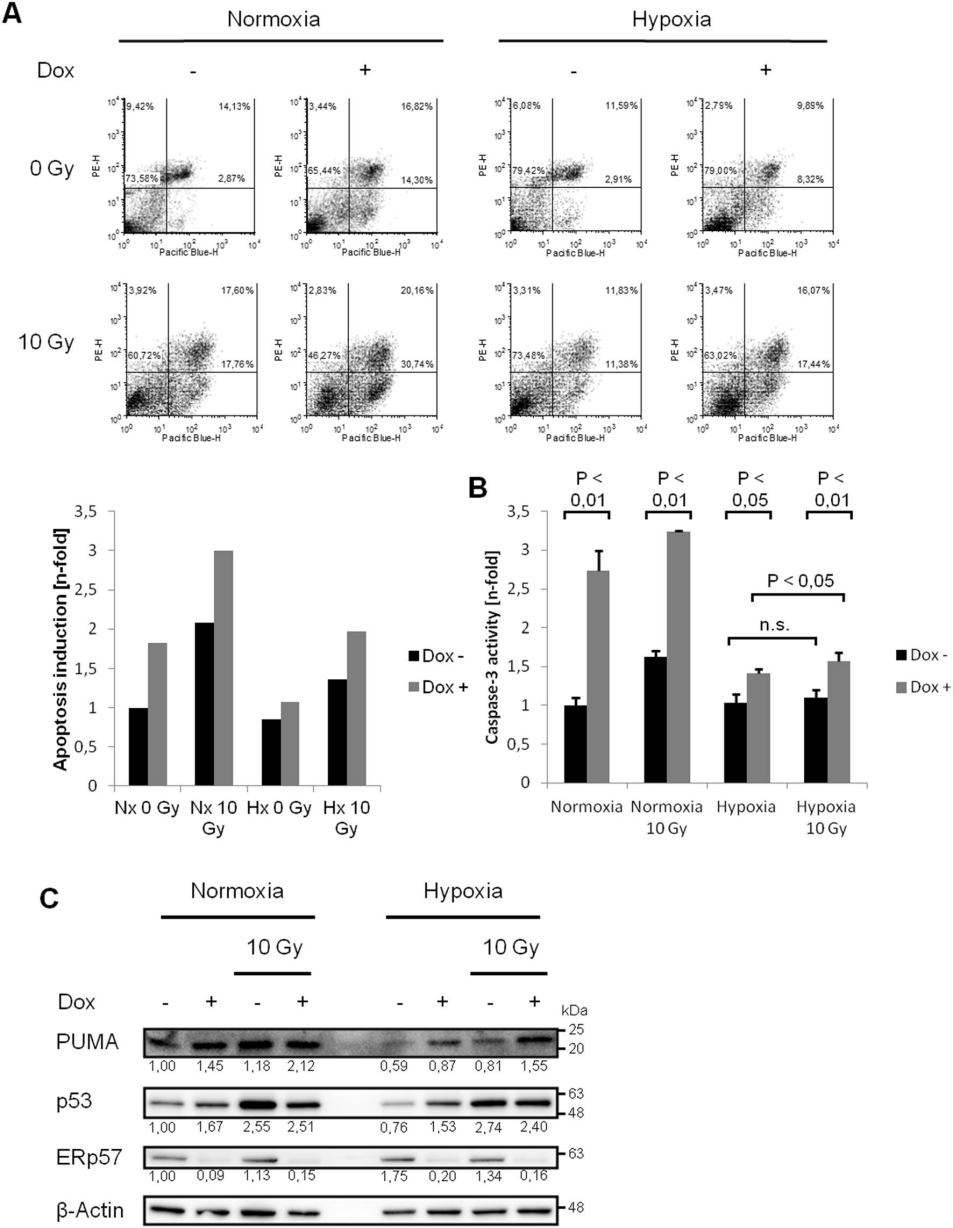
Depletion of ERp57 increases apoptosis in hypoxia. (**A**) Annexin/ propidium iodide staining was performed to detect early and progressed apoptosis after radiation with 10 Gy or ERp57 KD. Annexin was conjugated to Pacific Blue and thereby designated as „Pacific Blue-H “ in the charts. Propidium iodide as indicator for progressed apoptosis was designated as PE-H. The diagram shows one representative result out of three performed experiments. The induction of apoptosis due to the respective treatment was illustrated as n-fold induction to the untreated sample. Early and late apoptosis were summed as comprehensive apoptosis. (**B**) Caspase-3 activity was measured 96 h after KD and 48 h after radiation with 10 Gy. Cells were in hypoxia for 72 h. (**C**) After the same treatment as in (**B**) pro-apoptotic proteins p53 and PUMA were checked by Western blot.

In an earlier work, we demonstrated that apoptosis is elevated in HCT116 colon cancer cells when ERp57 is depleted^9^. Throughout this study, we used the same lentivirally-based, doxycycline (DOX)-inducible knockdown system. To cover growth conditions that are also found in solid tumors, we extended the experimental setup from normoxia (21% O_2_) to mild hypoxia (1% O_2_) and performed Annexin/ propodium iodide (Anx/PI) staining (Fig. 1A): Sole treatment of ERp57 KD or irradiation increased apoptosis albeit less pronounced than in normoxia. Combined treatment of ERp57 depletion with irradiation nearly doubled the apoptotic fraction compared to the untreated hypoxic sample. Similar results were obtained using caspase-3 activity as a second readout for apoptosis (Fig. 1B). Additional western blot analysis showed induced levels for p53 and PUMA after ERp57 KD supporting our apoptotic measurements (Fig. 1C). However, their protein levels were upregulated oxygen-independently while Anx/PI staining and capase-3 assay measured decreased apoptosis under hypoxia. This discrepancy is probably due to hypoxia-induced expression of anti-apoptotic factors like BCL2 that bind and inactivate PUMA, thereby reducing the apoptotic response without detectable differences in protein levels^14,15^.

### ERp57 is crucial for hypoxic cell proliferation and does not influence the HIF pathway

**Figure 2:**
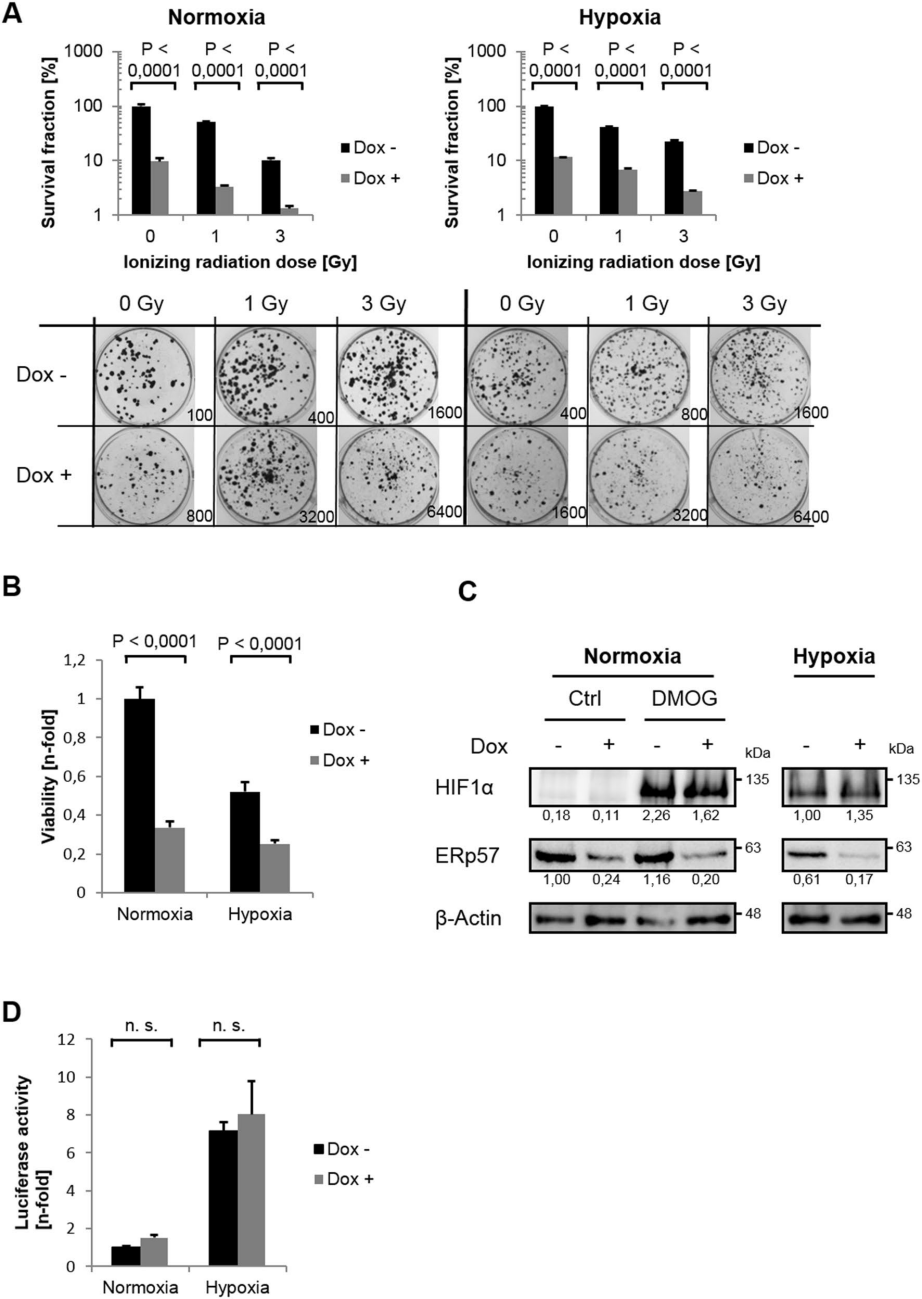
ERp57 promotes hypoxic proliferation, is radioprotective and does not effect HIF signaling. (**A**) For colony formation assay, cells were seeded supplemented with doxycycline and incubated under normoxic or hypoxic conditions. After 24 h, cells were irradiated with 0, 1 and 3 Gy and further incubated for 10 d before formed colonies were counted. Survival fraction was presented in bar graphs and representative images of colonies formed in 6 well plates are shown. The seeded cell number is indicated for each well. (**B**) MTT assay 96 h after ERp57 KD (n = 10). (**C**) Protein analysis of HIF1α 72 h after ERp57 KD and 24 h of hypoxia by western blot. As a positive control for HIF1α expression, normoxic cells were treated with 2 mM of prolyl-4-hydroxylase inhibitor dimethyloxalylglycine (DMOG). (**D**) HIF reporter gene assay. After co-transfection with pGLHIF1.3 (firefly luciferase, 3 × HRE) and pGL4.74 (renilla luciferase), ERp57 KD was induced. After 24 h in normoxia, cells were incubated for 48 h in normoxia/ hypoxia before cell lysis (n = 4).

To evaluate a long-term effect of the ERp57 KD in hypoxia, clonogenic assays were performed (Fig. 2A). As in normoxia, the depletion of ERp57 alone drastically reduced the survival fraction (SF) down to 10% in hypoxia and was thereby even more effective than irradiation with 3 Gy. The combination of KD and irradiation with 3 Gy revealed the strongest phenotype of 2.7% SF under hypoxic condition. Next, we performed MTT assays to examine cell viability (Fig. 2B). The lack of ERp57 compromised cellular energy levels independent of the oxygen level as indicated by a decrease of at least 50% in normoxia and hypoxia compared to the respective controls. We further investigated a potential influence of ERp57 KD on hypoxia-inducible factor (HIF), the master regulator of O_2_ homeostasis. As demonstrated by Western blot for HIF1-α (Fig. 2C) and by luciferase assay for total HIF activity (Fig. 2D), the function of HIF was not affected by deprivation of ERp57.

### PDPK1 depends on ERp57 but is expendable for cancer cell growth

**Figure 3:**
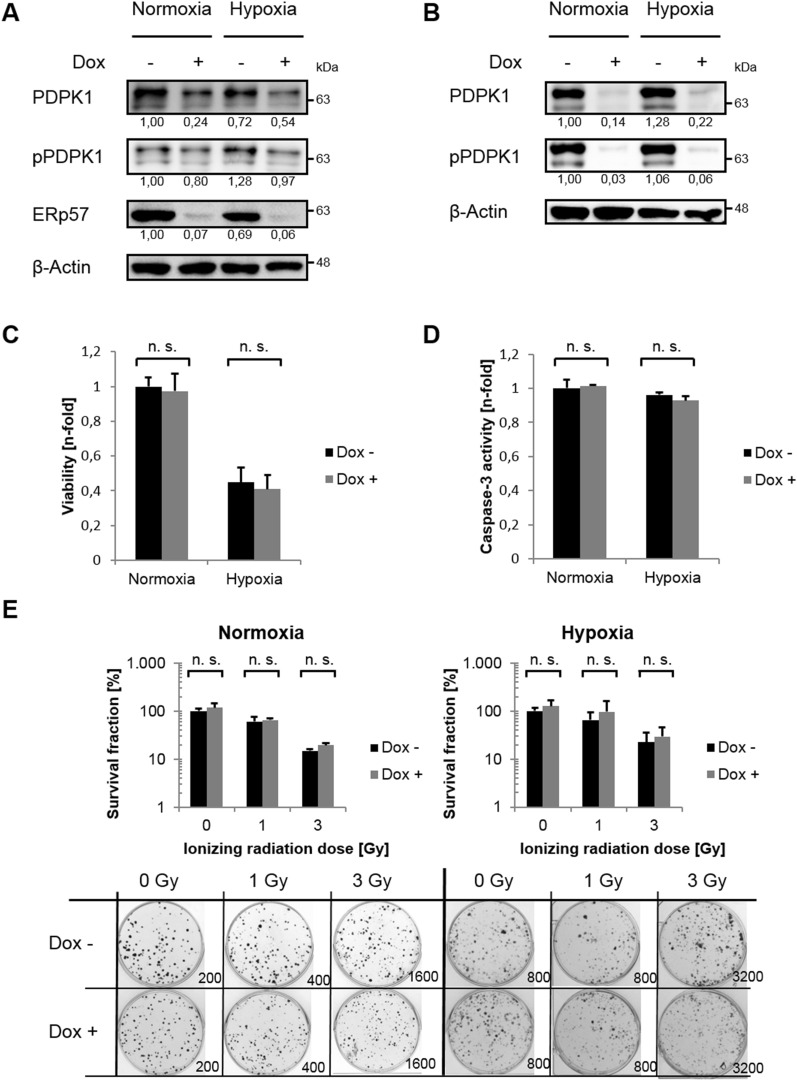
PDPK1 KD does not effect cancer cell growth. (**A**) Western blot analysis of PDPK1 after 96 h KD of ERp57. (**B**) Western blot analysis of inducible PDPK1 KD in HCT116 cells. (**C**) MTT viability assay. Cells were incubated 96 h after PDPK1 KD before analysis. (**D**) Apoptosis was analysed by Caspase-3 activity assay 96 h after PDPK1 KD. (**E**) Colony formation assay after PDPK1 KD. Irradiation with 0, 1 and 3 Gy took place 24 h after seeding. Formed colonies were counted 10 d after seeding. Survival fraction is presented in bar graphs and representative images of colonies formed in 6-Well plates are shown.

**Figure 4:**
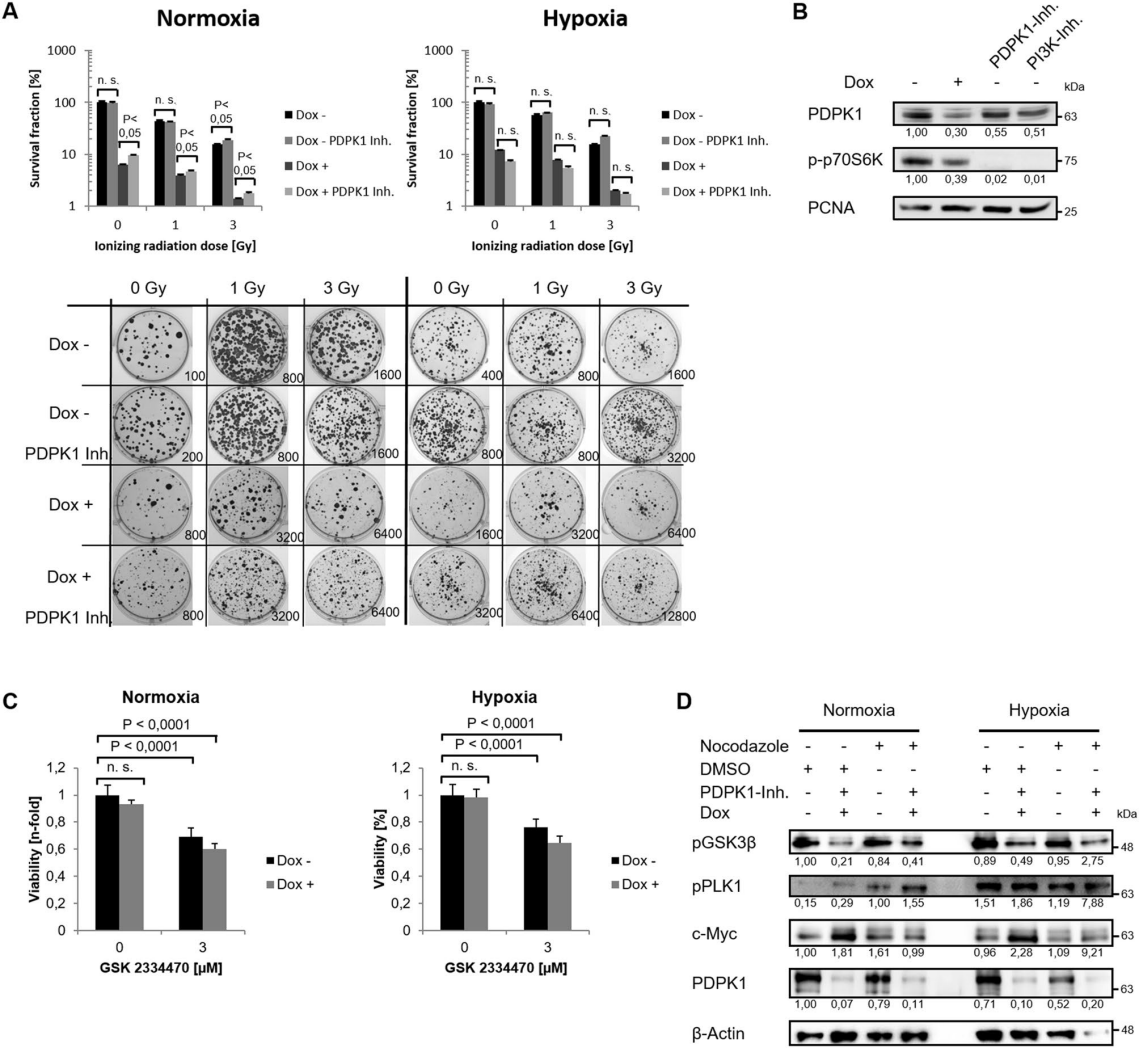
Combination of ERp57 KD and chemical inhibition of PDPK1 does not effect cancer cell growth. (**A**) Colony formation assay after ERp57 KD in combination with PDPK1 inhibitor GSK 2334470 (3 µM). KD induction and inhibitor treatment was carried out right after seeding. The cells were irradiated 24 h after seeding. Formed colonies were counted 10 d after seeding. Survival fraction is presented in bar graphs and representative images of colonies formed in 6-Well plates are shown. (**B**) Western blot analysis of PDPK1 target p70S6K after 96 h of ERp57 KD and chemical inhibition of PDPK1 (3 µM GSK 2334470) or PI3K (50 µM LY294002) in normoxia. PCNA was used as a loading control. (**C**) MTT viability assay after 96 h of PDPK1 KD in combination with PDPK1 inhibition. (**D**) Western blot analysis after 96 h of PDPK1 KD in combination with chemical PDPK1 inhibition (3 µM GSK 2334470). For additional analysis of pPLK1, cells were treated with 0.1 µg/ml nocodazole 16 h before lysis to achieve G2/M arrest. DMSO was used as a vehicle control.

We detected ERp57, originally described as a typical ER-resident protein, also in the cytoplasm of different cancer cell lines where it drives cell growth^9^. Hence, we started examining the phosphatidylinositol 3-phosphate kinase (PI3K)/ AKT pathway, one of the most well-known signal pathways that controls proliferation, cell cycle progression and metabolism in cancer cells^16^. First of all, we noticed changes in expression levels of the master kinase 3-phosphoinositide-dependent protein kinase 1 (PDPK1, PDK1) after depletion of ERP57: Total amount as well as the phosphorylated/ activated form of PDPK1 (pPDPK1) were both downregulated under normoxic and hypoxic conditions (Fig. 3A). To sort out if the lack of PDPK1 after ERp57 KD is responsible for the cell growth inhibition, we generated HCT116 cells with a doxycycline inducible small hairpin (shPDPK1) for PDPK1 and verified a KD efficiency of more than 90% for normoxia and hypoxia via immunoblotting (Fig. 3B). These cells were tested for cell viability, apoptosis and clonogenic survival after KD of PDPK1 but showed no phenotype at all (Fig. 3C–E). Instead, we observed a minor but not significant survival advantage when PDPK1 was missing (Fig. 3E). We compared this genetic approach with clonogenic survival assays of HCT116-shERp57 cells treated with the PDPK1 inhibitor GSK 2334470. However, we observed no altered proliferation after administration of the inhibitor either, only after ERp57 KD alone or in combination (Fig. 4A). Western blot analysis of activated/phosphorylated p70S6K, a PDPK1 downstream target, verified a functional PDPK1 inhibition for at least 4 days (Fig. 4B). As an additional control, the PI3K inhibitor LY294002 was used over the same time period. Further, we combined PDPK1 KD and chemical inhibition, analyzed cell viability by MTT assay and found a decreased viability in normoxia by about 40% and in hypoxia by about 35% (Fig. 4C). This combined treatment was also analyzed on protein level. Here, we observed a decrease of the PDPK1 target pGSK3β, while pPLK1 was unaffected and c-Myc levels were even upregulated (Fig. 4D).

### Deprivation of ERp57 impairs proliferation factors AKT, PLK1, ERK1/2 and c-Myc independently of cellular oxygen levels

**Figure 5:**
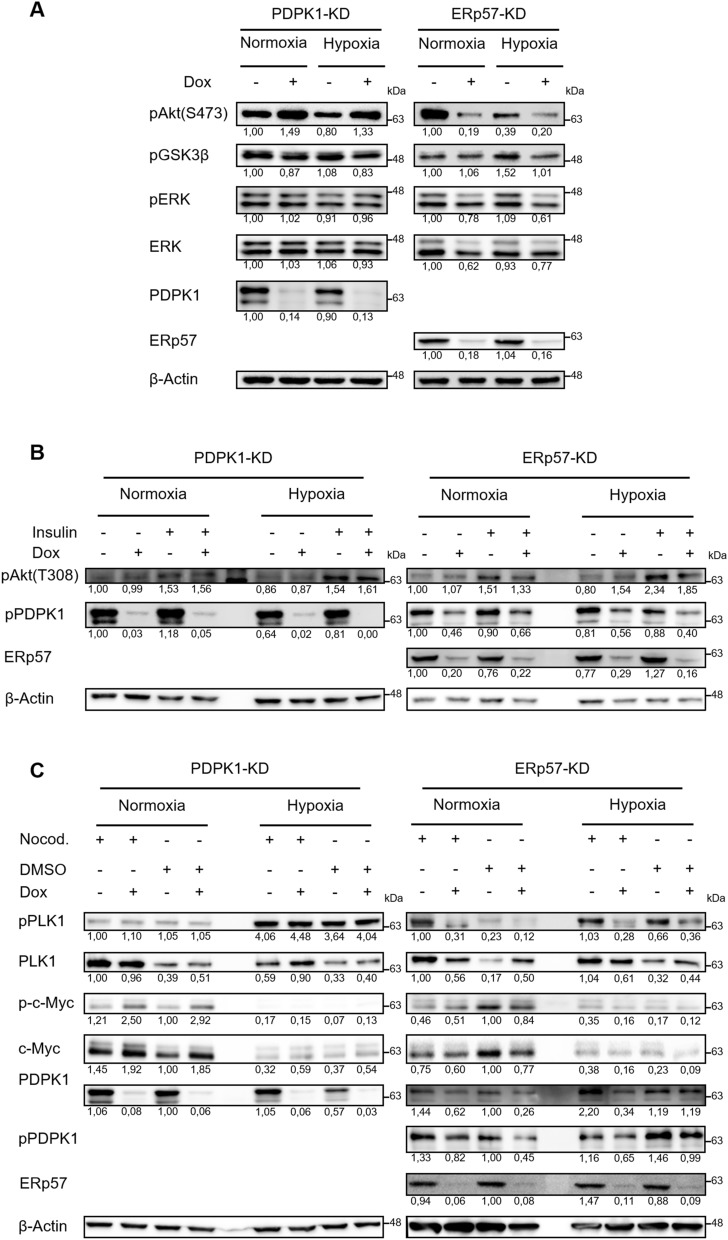
KD of ERp57, but not PDPK1, impairs multiple proliferation factors independently of cellular oxygen levels. (**A**)–(**C**) Comparison of different proliferation factors in ERp57 KD and PDPK1 KD cells 96 h after induction of KD. (**B**) Cells were treated with 1 mM insulin 3 h before lysis. (**C**) To analyze pPLK1 in the G2/M phase, cells were treated with 0.1 µg/ml nocodazole 16 h before lysis. DMSO was used as a vehicle control.

Since the decrease of PDPK1 protein after ERp57 KD did not cause the observed cell growth inhibition, we analyzed other PI3K downstream mediators by western blot: While depletion of PDPK1 triggered phosphorylation of AKT at Ser473 and did not change its phosphorylation status at Thr308, KD of ERp57 instead minimized active AKT (pAKT-Ser473/ Thr308) under normoxic and hypoxic conditions, however more severe for pAKT-Ser473. We further noticed a reduced phosphorylation of AKT-target GSK3β under hypoxia (Fig. 5A, B). In addition, we tested the MAPK/ ERK signal pathway and could show a moderate downregulation of total ERK1/2 and pERK1/2 after depletion of ERp57 (Fig. 5A). As we noticed in our previous study a G2 arrest after KD of ERp57^9^, we also looked into polo-like kinase 1 (PLK1) and oncogene c-Myc, both important factors for cell cycle progression and cell growth^17,18^. Comparing KD of PDPK1 and ERp57, we detected considerable differences in their expression/ activation levels (Fig. 5C): The lack of ERp57 resulted in a downregulation of total PLK1 and pPLK1 independent of the oxygen level, whereas the KD of PDPK1 rather increased the total amount of PLK1 in hypoxia but had no effect on its phosphorylation status (compare nocodazole treated samples). As for PLK1, we noticed a similar downregulation for c-Myc / p–c-Myc when ERp57 was depleted, even though less pronounced in hypoxia due to lesser expression levels of c-Myc under oxygen deprivation. In contrast, KD of PDPK1 even triggered the upregulation of c-Myc / p–c-Myc (compare non-nocodazole treated samples).

**Figure 6:**
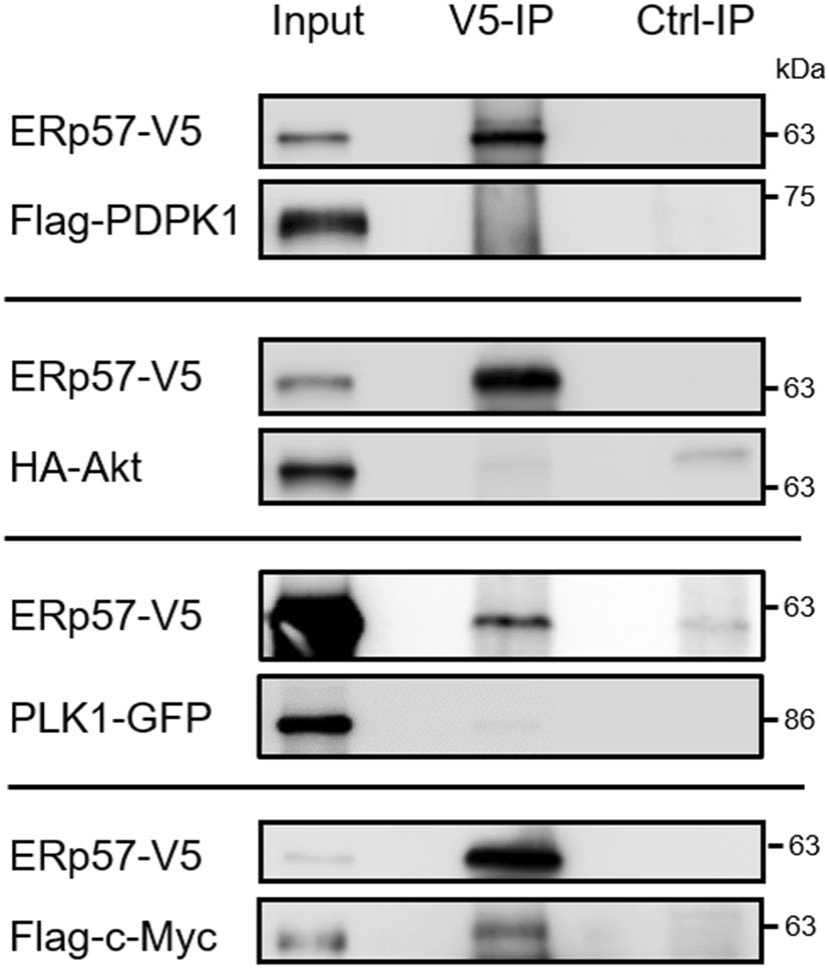
Analysis of protein–protein interaction for ERp57 with PDPK1, AKT, PLK1 and c-Myc. Plasmid pe-N1-ERp57-V5 was co-transfected with vectors CMV-3xFlag-hPDPK1, pCDNA3-HA-AKT1, pEYFP-PLK1 or pCMV4a-Flag-c-Myc into HEK293T cells. For all IP experiments, ERp57 was pulled with an anti-V5 antibody (V5-IP) and complex formation with PDPK1, AKT, PLK1 and c-Myc was determined by western blot analysis using anti-FLAG, anti-HA, anti-GFP and anti-c-Myc antibodies, respectively. 1–3% of total cell lysate was loaded as input.

As the depletion of ERp57 had a strong impact on PLK1, PDPK1, AKT and c-Myc, we further checked whether these proteins show a protein complex formation with ERp57 by CO-IP experiments (Fig. 6). After co-overexpression in HEK293T cells, only c-Myc could be identified as a potential protein interaction partner of ERp57. For the other targets, either no signal in the IP lane was detectable (PDPK1 and AKT) or the signal did not exceed the background level visible in the control IP (PLK1).

## Discussion

In previous work, we and others were able to detect a minor fraction of ERp57 in the cytosol^9,12,13^. Consequently, the impact of ERp57 depletion on the PI3K pathway and other proliferation factors described in this study suggested a direct or indirect protein interaction with cytoplasmic ERp57. Except for c-Myc, our Co-IPs could not confirm this for PDPK1, AKT or PLK1 although we cannot rule out protein complexes of ERp57 that were too weak or too transient to detect under the chosen conditions. Besides a direct involvement in proliferation from the cytoplasmic site, it was also conceivable that ERp57 promotes cell proliferation indirectly from the ER compartment: Since KD of ERp57 triggers ER stress via the PERK pathway and results in global protein shutdown^9^, this could also have caused attenuation of the affected growth factors. Notably, this hypothesis was tested recently in a study where the additional depletion of PDI inhibited the PERK-related UPR completely but did not alter the detrimental effect on proliferation after ERp57 KD alone^11^. Finally it is also possible that nuclear ERp57, acting as a transcriptional cofactor^19^, takes part in the expression of proliferation related pathways, as several studies could already show ERp57s influence on genes which are involved in intracellular traffic and stress responses^19–21^. However, since we have been not able to detect nuclear ERp57 in various cell lines^9^, we decided not to conduct gene expression analysis in this study.

PDPK1 is called a master kinase of the PI3K pathway for its outstanding role as an upstream kinase capable of phosphorylation and activation of a whole set of AGC kinases (e.g. AKT, S6K, RSK, PKC and SGK) that are involved in cell proliferation^22^. Beyond AKT, PDPK1 was further reported to trigger the well-studied oncogene c-Myc via phosphorylation of PLK1^23^. Therefore, our initial findings of a severe growth inhibition pointed straight to PDPK1 as the main effector of the ERp57 KD since not only PDPK1, but also PLK1 and c-Myc were all depleted at the same time. The reduction in PLK1 could also explain the former observation of an increased G2 arrest after KD of ERp57^9^. Unexpectedly, neither a chemical inhibition of PDPK1 with 3 µM compound GSK 2334470 (IC_50_ ≈ 10 nM) nor a generated cancer cell line with an inducible KD of PDPK1 (> 90% KD) did mimic the phenotype of ERp57 KD. Even though the combined treatment of inhibitor and KD finally effected tumor cell viability, the reduction did not exceed 40%. At first glance these results were hard to explain regarding the claimed central role for PDPK1. However, in line with our data is a former in vivo study reporting that an inducible PDPK1 KD of 90% efficiency failed to suppress tumor formation in three different mouse models of PTEN-deficient cancer^24^. The authors demonstrated that the residual PDPK1 protein was sufficient to uphold the PI3K pathway by continuous activation of AKT at Thr308. Furthermore, various cancer cell lines were able to bypass the PI3K pathway via phosphorylation of AKT by Ca^2+^/ calmodulin dependent protein kinase kinase (CAMKK2)^25^. Based on these and our results, it is at least questionable whether cancer patients would benefit from a monotherapy with a potent PDPK1 inhibitor molecule and it seems more advisable to target AKT directly.

In fact, numerous AKT inhibitors are in clinical development and passed phase 1/2 trials^26^. However, potent cancer drugs that only target one single pathway are likely to fail their clinical expectations due to the frequent occurrence of drug resistance. Redundant pathways can take over and bypass the blockade through rewiring the oncogenic cellular network^27^. Here, in search of new druggable targets, we tested the relevance of ERp57 for tumor growth in the context of oxygen deprivation. Insufficient oxygenation contributes to tumor progression and triggers stabilization of HIFs that mediate resistance to chemo- and radiotherapy^4^. Even though KD of ERp57 did not affect HIF activity, we presented compelling data that the depletion of ERp57 severely inhibits proliferation by downregulating a set of notorious oncogenes under moderate hypoxia (1% O_2_). Notably, we and others demonstrated earlier that ERp57 is also essential for sufficient mTORC1 activity^9,12^. Considering the size of the PDI family with more than 20 group members and a redundance in their substrate spectrum^28^, it was quite surprising that so many proliferation factors are compromised at once by the KD of one single family member. Although the number of ERp57`s interaction partners in various cell compartments continuously grow^29^, further research work is needed to reveal the complete network of ERp57 and the exact molecular mechanisms that explain such an impact on so many different cellular targets under normoxic and hypoxic conditions. In terms of ERp57 as a target for combination therapy, strong radio- and chemosensitizing effects were observed in cancer cells that were irradiated/ etoposide-treated under ERp57 KD in normoxia^9^. Here, these results were mirrored for irradiation treatment under oxygen deprivation indicating that inhibition of ERp57 has the potential to overcome hypoxic-induced tumor radioresistance. Interestingly, a role of nuclear ERp57 in DNA damage repair was already suggested in earlier studies^30,31^. PDI and ERp57 were also proposed as potential biomarker in ovarian cancer, since high expression correlates with poor patient outcome^32^. Depletion of ERp57 led to a severe impairment of the PI3K pathway which is frequently mutated and overactive in colorectal carcinoma, implying a rational strategy to overcome PI3K triggered, unregulated proliferation by inhibiting ERp57^33^. Therefore, and regarding numerous reports of ERp57′s involvement in other cancer models^30,32,34–38^, we postulate that this ERp57-dependend effect on cell growth, chemo- and radiosensitivity is not cell-type specific and affects cancer cells beyond colorectal and mammary origin used in our studies.

To our knowledge, no specific ERp57 inhibitor is available so far. But regarding the high structural similarity of ERp57 with its close homologue PDI and several reports of well tolerated PDI inhibitors in preclinical studies^39–43^, there is reasonable hope that a chemical (or genetic) inhibition of ERp57 could turn out to be more effective in mono- and combination therapy than single targeting inhibitors and could minimize toxicity in non-cancer tissue of patients during treatment.

## Materials and methods

### Antibodies and reagents

Anti-ERp57 (ab10287) and Anti-pAkt (Thr308) (ab194875) antibodies were from Abcam (Cambridge, UK). Anti-β-Actin (A2103) antibody, PDPK1 inhibitor GSK2334470 (SML0217), Nocodazole (M1404) and human Insulin (91077C) were from Sigma-Aldrich (Munich, Germany). Anti-p53 (GTX102965) antibody was from GeneTex (Hsinchu City, Taiwan). Anti-PUMA (ABC158) antibody was from Millipore (Burlington, MA, USA). Anti-HIF1α (610,959) was from BD Transduction Laboratories (San Jose, CA, USA). Anti-c-Myc (#13,987), Anti-ERK1/2 (#4695), Anti-GFP (#2956), Anti-HA-Tag (#3724), Anti-pAkt (Ser473) (#4060), Anti-p–c-Myc (#13,748), Anti-pERK1/2 (#4370), Anti-pGSK3β (#5558), Anti-PDPK1 (#3062), Anti-PLK1(#4513), Anti-p-p70S6K (#9205), Anti-pPDPK1 (#3438), Anti-pPLK1 (#9062), Anti-PCNA (#2586), Anti-V5-Tag (#13,202) antibodies and PI3K inhibitor LY294002 (#9901) were obtained from Cell Signaling Technology. DMOG (BML-EI347) was purchased from Enzo Life Sciences (Lörrach, Germany).

### Cell lines, cell culture, hypoxic treatment and cell cycle synchronization

For all experiments colon carcinoma cell line HCT116 (ATCC, CCL-247) was used. Cells were cultured in McCoy’s 5A medium (Lonza, Basel, Switzerland) supplemented with 10% FBS and antibiotics. For normoxic conditions cells were incubated with 21% O_2_ and 5% CO_2_. For hypoxic conditions cells were kept in hypoxic incubator (Toepffer Lap Systems, Göppingen, Germany) with 1% O_2_ and 5% CO_2_. For cell cycle synchronization in G2/M phase cells were treated with 100 ng/ml nocodazole 16 h before lysis. Controls were treated with DMSO as vehicle. Treatment with 1 mM insulin took place 3 h before lysis. All experiments were performed with mycoplasma-free cells.

### Plasmid construction, transfections, lentiviral production and knockdown induction

To introduce a C-terminal V5-tag into ERp57 (pe-N1-ERp57-V5), a modified QuickChange protocol was used^44^ to perform a PCR using pEGFP-N1-ERp57 as the template^9^ and primers ERp57-V5-for (5′-AAGGCACAGGAGGATCTC GGTAAGCCTATCCCTAACCCTCTCCTCGGTCTCGATTCTACGTAGCCACCGGTC GCCACCATGGTGAGC -3′) and ERp57-V5-rev (5′ GAGATCCTCCTGTGCCTT CTTCTTCTTCTTGGGTTTTTCTTCTTGAATTACAGGGGGG-3′). For transient transfection, ViaFect (Promega Corporations, Mannheim, Germany) was used as manufacture’s protocol suggests. Lentiviral production was conducted as described before^11^. Briefly, for transduction 2 × 10^5^ HEK293T cells were incubated with 2 × 10^6^ transduction units for 24 h in attendance of 8 µg/µl polybrene. Transduced HCT116 cells were selected by treatment with 2 µg/ml puromycin for 7 days. pLKO.1-shRNA-ERp57 tet-on (tetracycline inducible) contained the sequence 5′-GGAATAGTCCCATTAGCAAAG-3′ of ERp57 mRNA (GenBank acc. No. NM_005313). pLKO.1-shRNA-hPDPK1 tet-on contained the sequence 5′-CAAAGTTCTGAAAGGTGAAAT-3′ of hPDPK1 mRNA (GenBank acc. No. NM_001261816). Gene KD was induced by addition of 250 ng/ml doxycycline (DOX) to the medium.

### Apoptosis analysis

Apoptosis were analyzed by caspase-3 enzyme activity assay and Annexin/ propidium iodide (Anx/PI) staining. Caspase-3 activity assay was performed as described previously^45^. AMC fluorescence was measured every 10 min at an excitation of 360 nm and emission of 460 nm over 2.5 h in a fluorescence reader (Synergy HT, Biotek, Bad Friedrichshall, Germany). For analysis, replicate values of a single timepoint in the linear range of the reaction were plotted. Measurement of apoptosis by flow cytometry with Anx/PI staining was performed as described previously^9^. Shortly, for staining 1 × 10^5^ cells were incubated with 80 µg/ml PI and 9.6 µg/ml Annexin V conjugated to PacificBlue (No. 640917, Biolegend, San Diego, CA, USA) in Annexin V binding buffer (No. 422201, Biolegend) for 15 min at room temperature. 10 000 cells were analyzed on a FACS Canto II (BD Biosciences, Franklin Lakes, NJ, USA) per sample. Data were analyzed using FCS Express 4 Flow software (De Novo Software, Los Angeles, CA, USA).

### Cell viability

Cell viability was analyzed by MTT assay. Therefore, 2 000 cells were plated in 96-well plates. After 96 h of knockdown induction cells were incubated with MTT for 4 h and afterwards lysed. Absorption was measured with fluorescence reader (Synergy HT, Biotek) at 540 nm.

### Clonogenic survival assay

To check for long term cell survival colony formation assay (CFA) was performed as described previously^46^. To enable formation of colonies under irradiation, cells were irradiated with no more than 3 Gy. Briefly, 100 to 6400 cells were plated on coated 6-well plates. For coating 9.8 ml DPBS, 200 µl rat collagen (Biozol, Eching, Germany) and 5 µM NaOH were used. After 24 h cells were irradiated with 1 or 3 Gy. 9 d later cells were fixed using para-formaldehyde and stained with Coomassie brilliant blue (containing 0.1 Coomassie blue, 5% acetic acid, 45% methanol). For analysis, survival fraction (SF) was determined by dividing number of formed colonies by number of plated cells in respect of plating efficiency (PE) (SF = number of formed colonies/ number of plated cells/ PE). For PE following formula was used: PE = number of formed colonies/ number of plated cells).

### Luciferase reporter gene assay

5 × 10^4^ cells were seeded in 24-well plates. After 24 h, cells were transfected with 500 ng pGLHIF1.3^47^ and 100 ng pGL4.74 renilla luciferase (#E6921, Promega) per well. After incubation for further 72 h, cells were lysed and prepared using the Dual Glow luciferase assay kit (Promega). Firefly and renilla luciferase were measured using the GloMax detection system (Promega) and normalized to renilla values to exclude variations in transfection.

### SDS PAGE and Western blotting

Whole cell extracts were prepared using RIPA buffer. Proteins were separated in a 7.5% polyacrylamide gel and subsequently transferred onto a PVDF membrane. 5% skimmed milk in TBS-T (50 mM Tris/HCl, 150 mM NaCl, 0.5% Tween-20, pH 7.2) were used to block membrane against unspecific antibody binding. Incubation with antibodies were performed as manufactures recommended. To detect secondary antibodies, ECL kit (34,095, Thermo Fisher Scientific, Waltham, MA, USA) and FX7 chemo luminescence documentation system (Peqlap, Erlangen, Germany) were used. Densitometry was used to quantify protein band intensity using software IMAGEJ. After normalization to each loading control, samples were compared to the untreated sample (set to 1).

### Co-immunoprecipitation

6 × 10^5^ HEK293T cells were seeded in a 6 well plate and co-transfected with 2–3 µg plasmid of pe-N1-ERp57-V5 and 2–3 µg of plasmid pcDNA3-HA-AKT1 (Plasmid #73,408, Addgene), pEYFP-PLK1 (Plasmid #39,843, Addgene), pRP[Exp]mCherry-CMV > 3xFlag-hPDPK1 (= CMV-3xFlag-hPDPK1, #VB180820-1170jnv, VectorBuilder) or pCMV4a-Flag-c-Myc (Plasmid #102,625, Addgene). After 72 h of incubation, cells were lysed in caspase lysis buffer (Tris (pH 7.3) 50 mM, NaCl 150 mM, NP-40 1% (v/v)) and incubated for 4 h at 4 °C with V5 antibody. As a control IP, lysate without V5 antibody was used. Afterwards, the lysate was incubated for 1 h with protein S and G magnetic beads (Cell Signaling Technologies, Danvers, USA). Beads were washed 4 × in caspase lysis buffer and finally transferred to SDS-sample buffer containing DTT and were heated at 95 °C for 10 min before loading on a 7.5% polyacrylamide gel.

### Statistical analysis

All experimental results were confirmed in at least 3 independent experiments except for Figs. 4B–D and 6 that were confirmed in two independent experiments. In graphs with bars at least three independent samples were taken unless it is otherwise described. Bars represent the mean of samples plus standard deviation (SD) of one representative experiment. For comparison two-way ANOVA by GraphPad Prism 7 (La Jolla, CA, USA) was applied with post hoc Bonferroni’s test. Specific P value is stated in the figures.

## Supplementary Information

Supplementary Information

## Data Availability

The data that support the findings of this study are available from the corresponding author upon reasonable request.
