# Supplementary material for: In oxygen-deprived tumor cells ERp57 provides radioprotection and ensures proliferation via c-Myc, PLK1 and the AKT pathway
Source: Sci Rep. 2021 Mar 30;11:7199. doi: 10.1038/s41598-021-86658-5 (PMC8009878; doi:10.1038/s41598-021-86658-5)
Supplement: Supplementary file 1 — Supplementary Information [file 41598_2021_86658_MOESM1_ESM.pptx]

## Slide 1
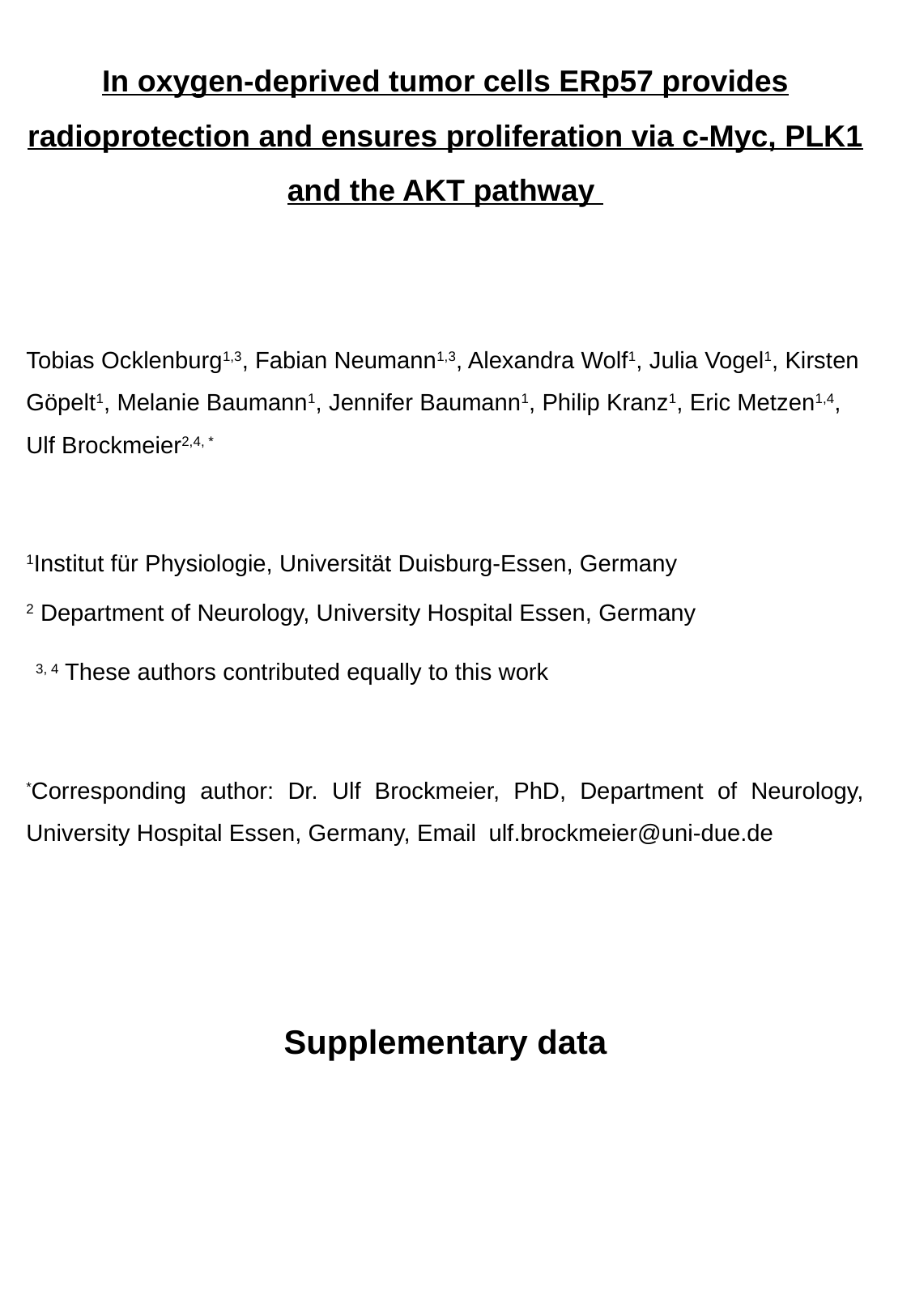

In oxygen-deprived tumor cells ERp57 provides radioprotection and ensures proliferation via c-Myc, PLK1 and the AKT pathway
Tobias Ocklenburg1,3, Fabian Neumann1,3, Alexandra Wolf1, Julia Vogel1, Kirsten Göpelt1, Melanie Baumann1, Jennifer Baumann1, Philip Kranz1, Eric Metzen1,4, Ulf Brockmeier2,4, *
1Institut für Physiologie, Universität Duisburg-Essen, Germany
2 Department of Neurology, University Hospital Essen, Germany
3, 4 These authors contributed equally to this work
*Corresponding author: Dr. Ulf Brockmeier, PhD, Department of Neurology, University Hospital Essen, Germany, Email ulf.brockmeier@uni-due.de
Supplementary data

## Slide 2
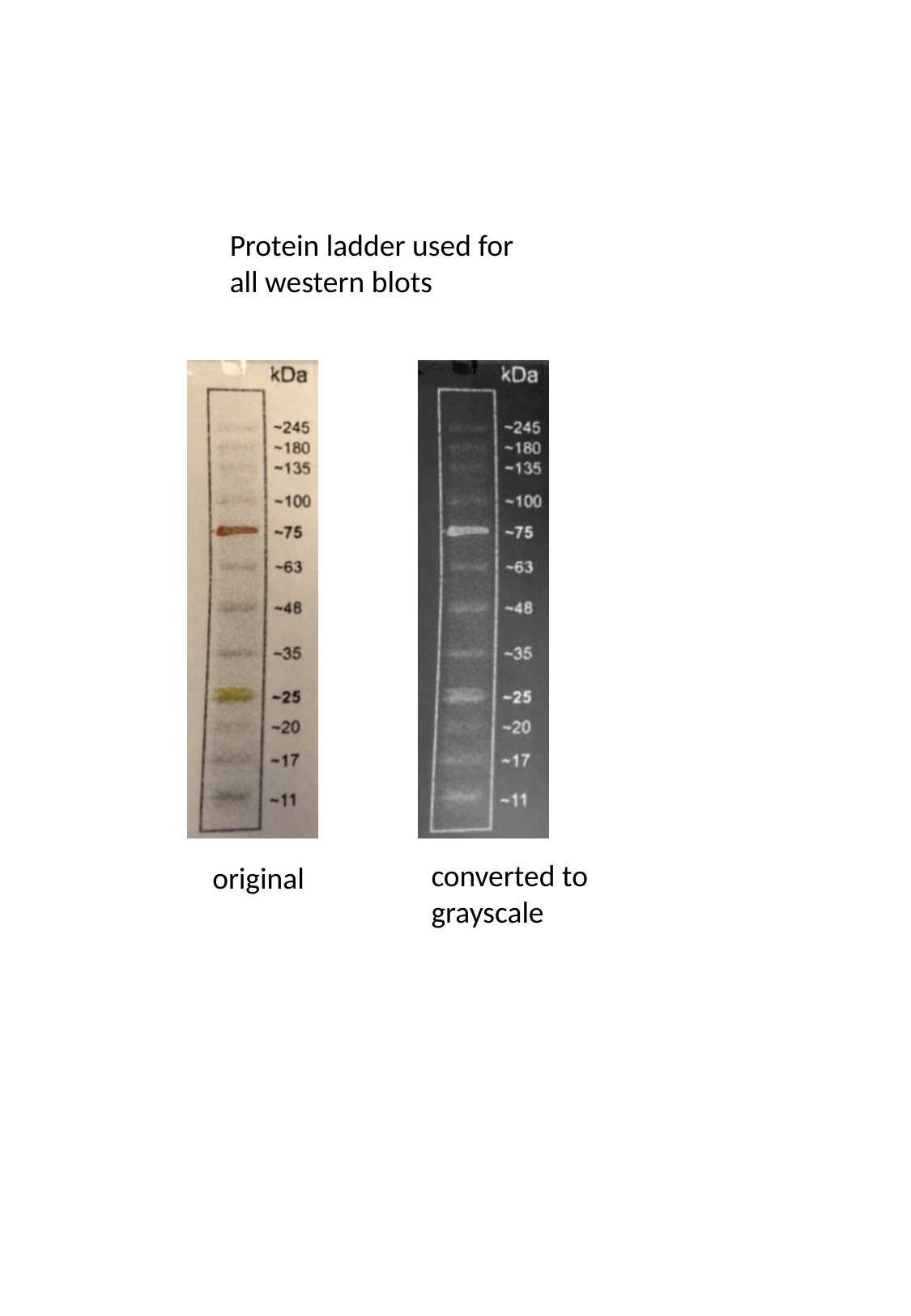

Protein ladder used for
all western blots
converted to
grayscale
original

## Slide 3
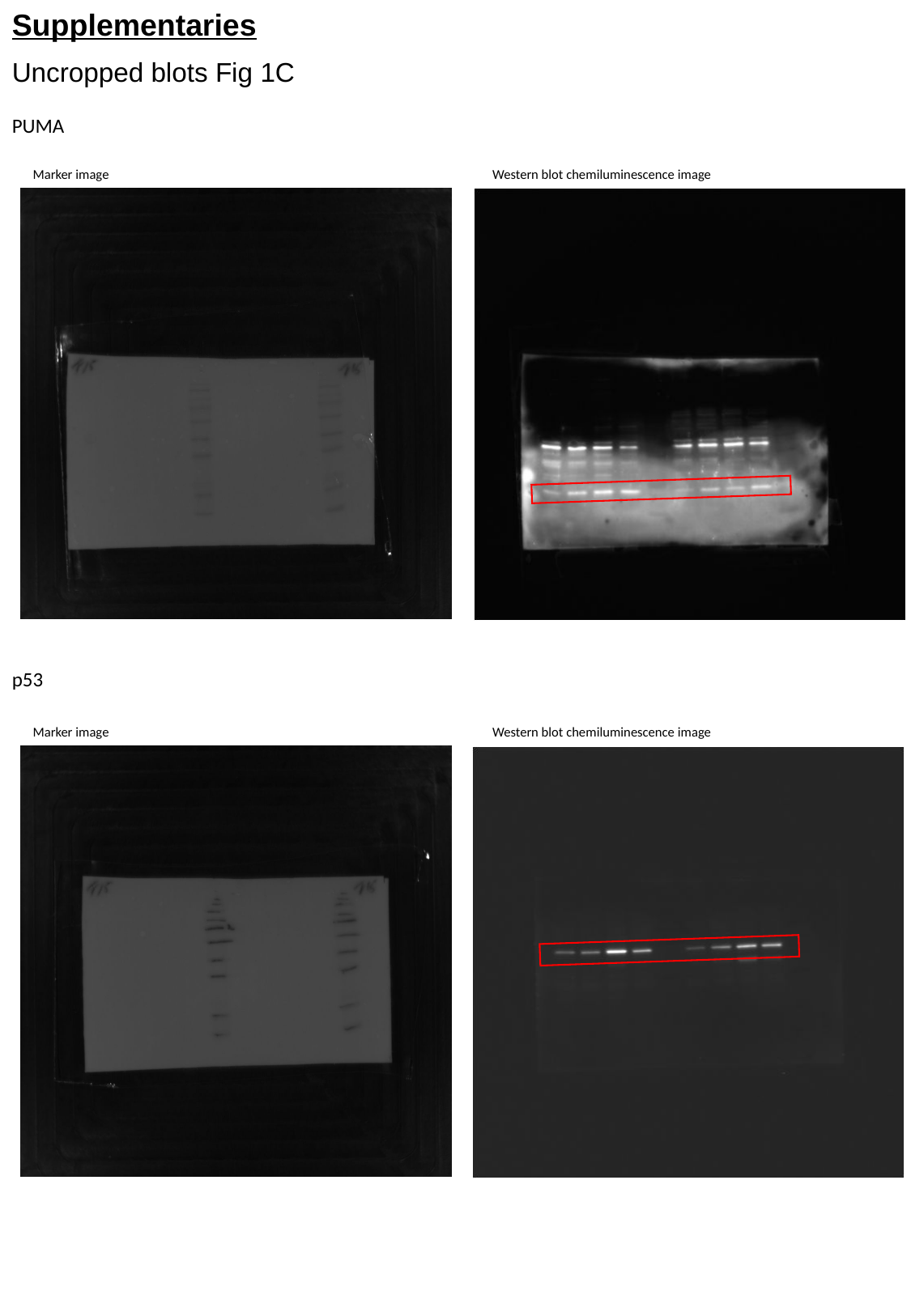

Supplementaries
Uncropped blots Fig 1C
PUMA
Marker image
Western blot chemiluminescence image
Western blot chemiluminescence image
p53
Marker image

## Slide 4
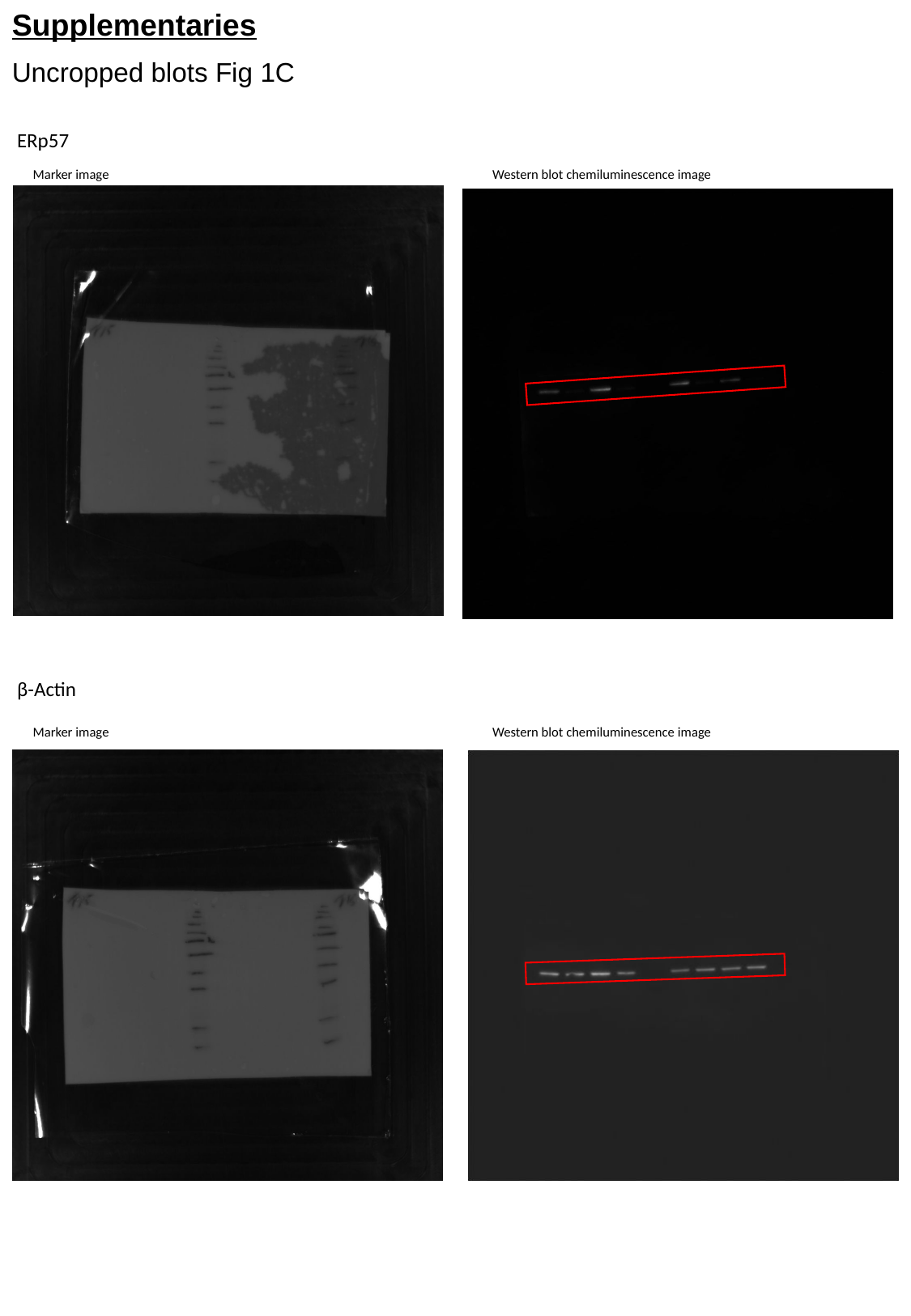

Supplementaries
Uncropped blots Fig 1C
ERp57
Marker image
Western blot chemiluminescence image
Western blot chemiluminescence image
β-Actin
Marker image

## Slide 5
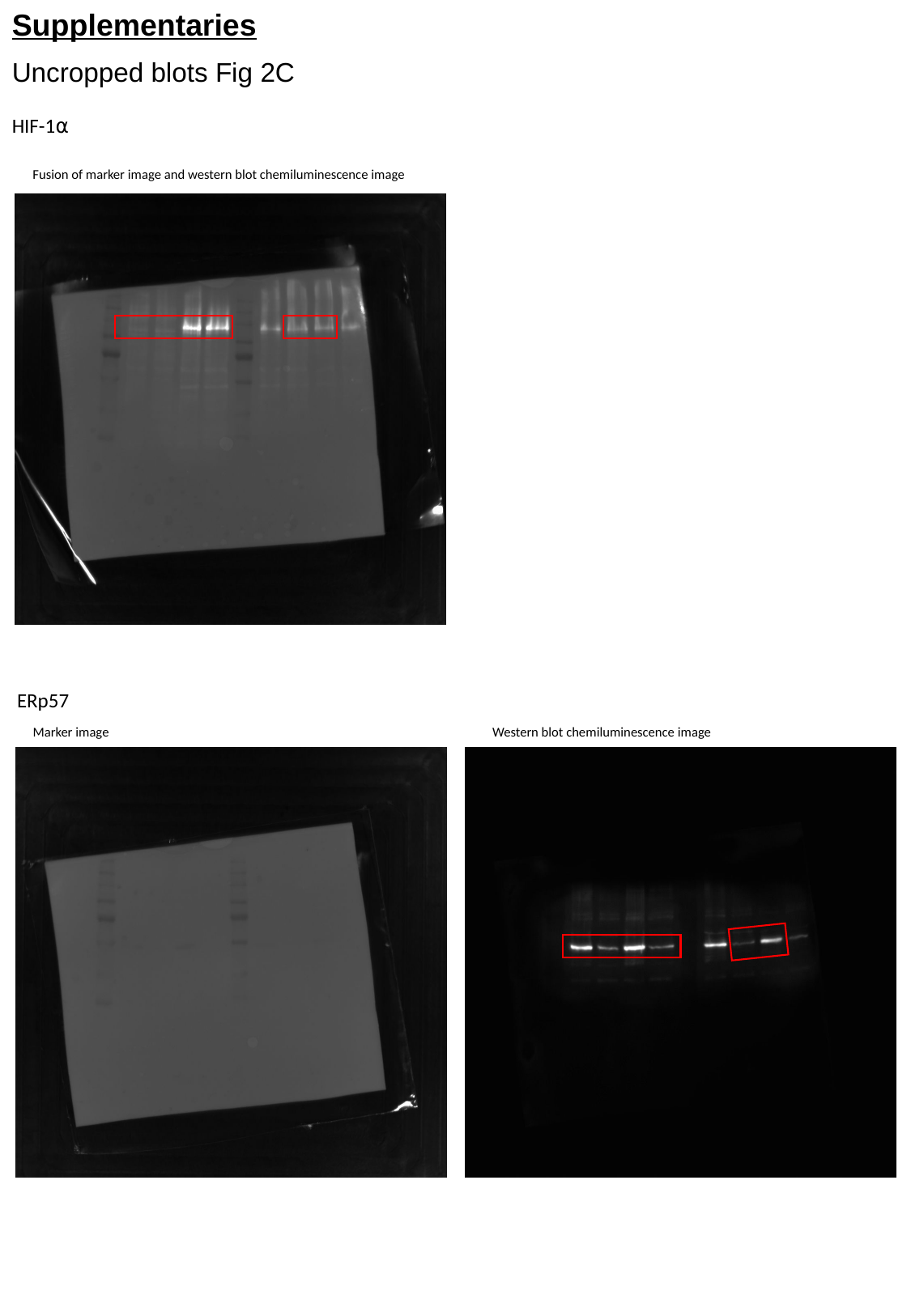

Supplementaries
Uncropped blots Fig 2C
HIF-1⍺
Fusion of marker image and western blot chemiluminescence image
ERp57
Marker image
Western blot chemiluminescence image

## Slide 6
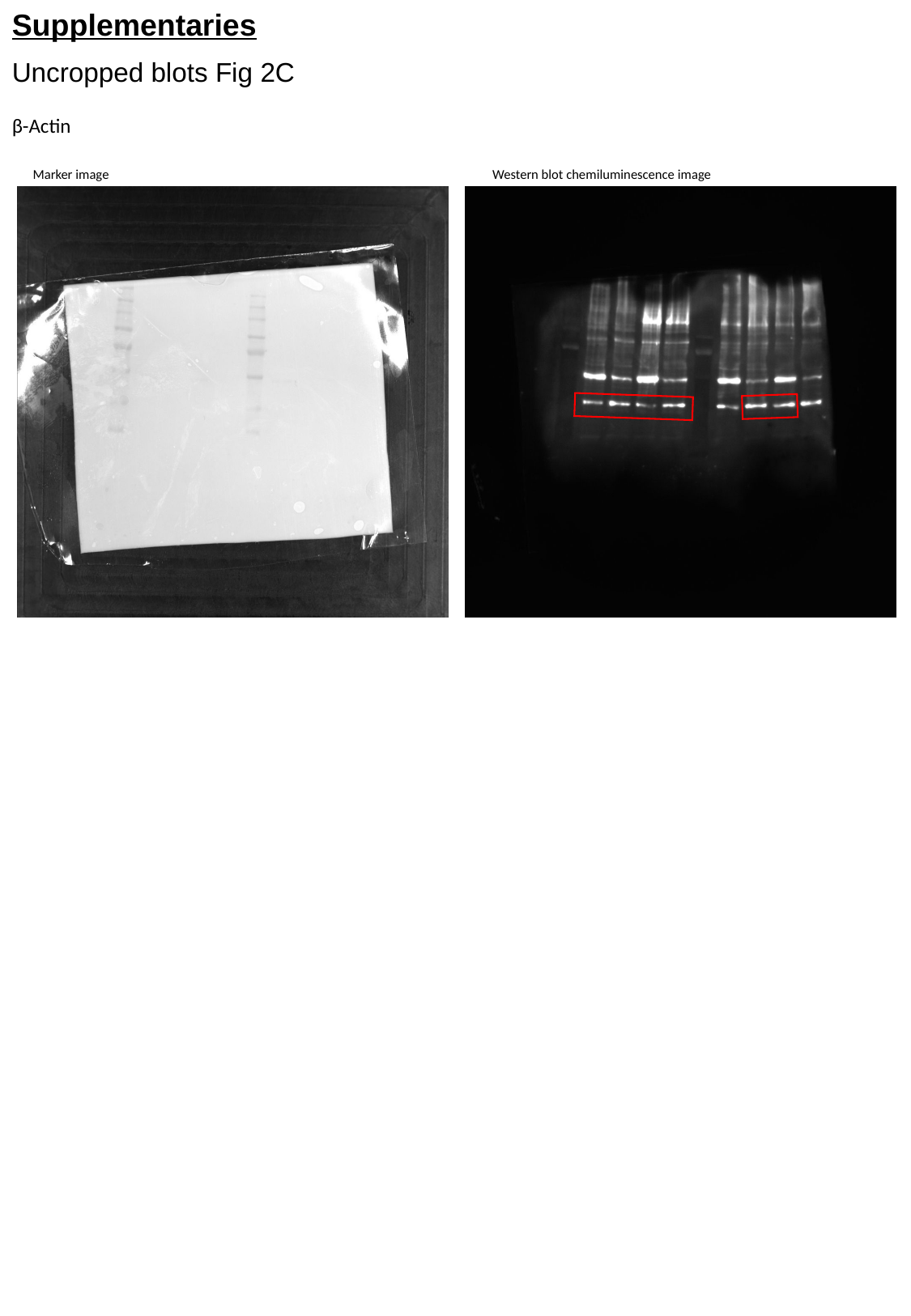

Supplementaries
Uncropped blots Fig 2C
β-Actin
Marker image
Western blot chemiluminescence image

## Slide 7
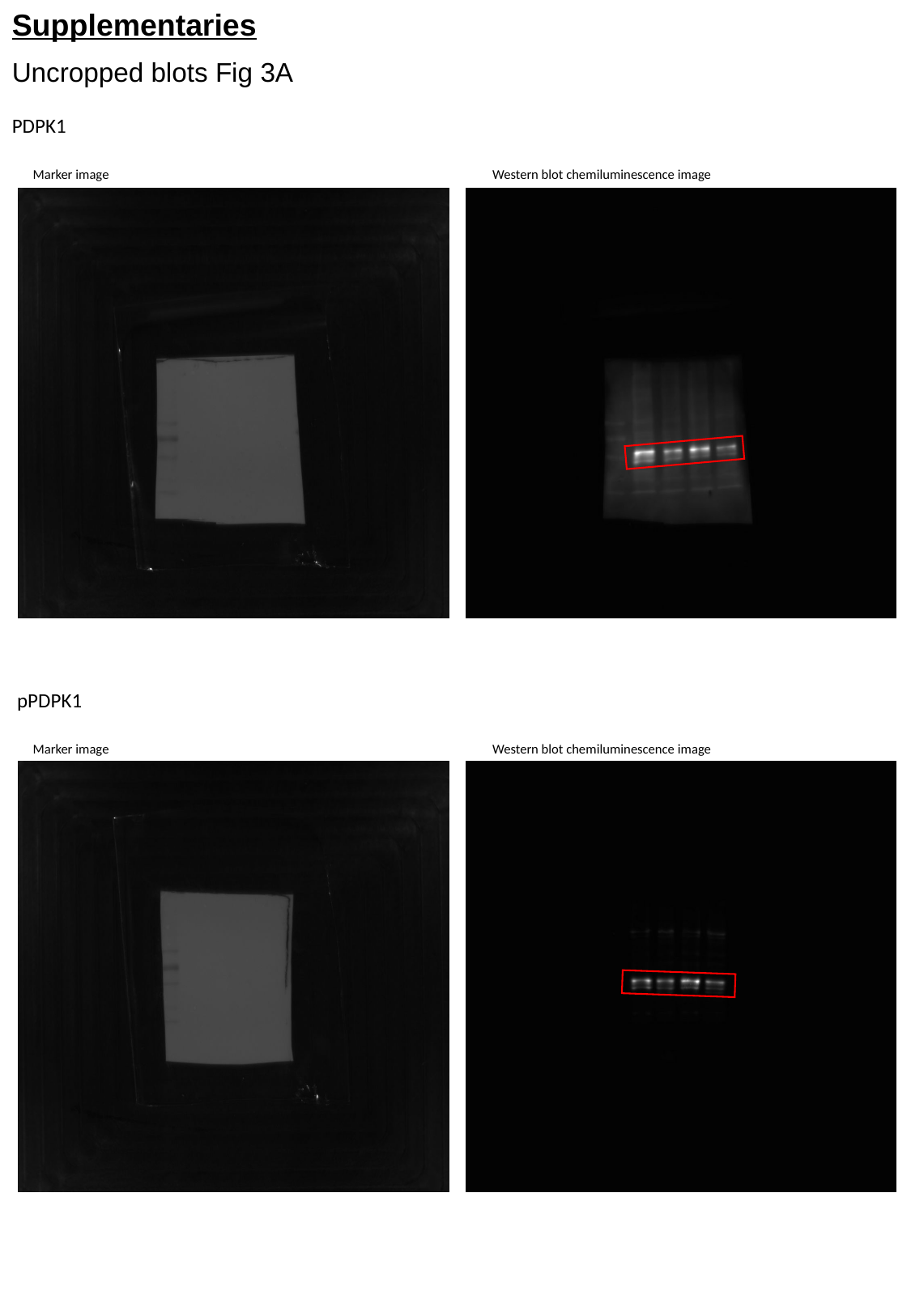

Supplementaries
Uncropped blots Fig 3A
PDPK1
Marker image
Western blot chemiluminescence image
pPDPK1
Marker image
Western blot chemiluminescence image

## Slide 8
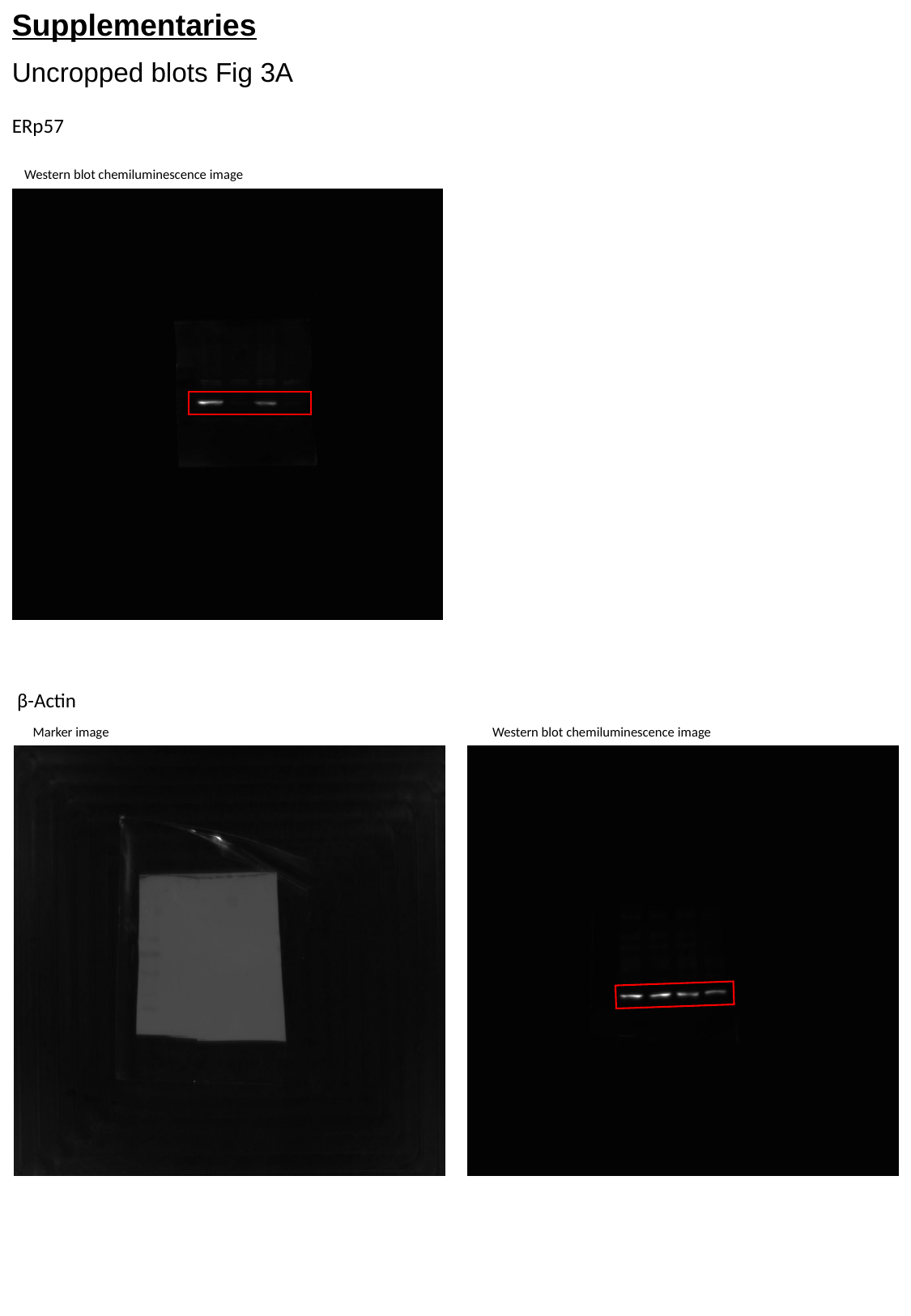

Supplementaries
Uncropped blots Fig 3A
ERp57
Western blot chemiluminescence image
β-Actin
Marker image
Western blot chemiluminescence image

## Slide 9
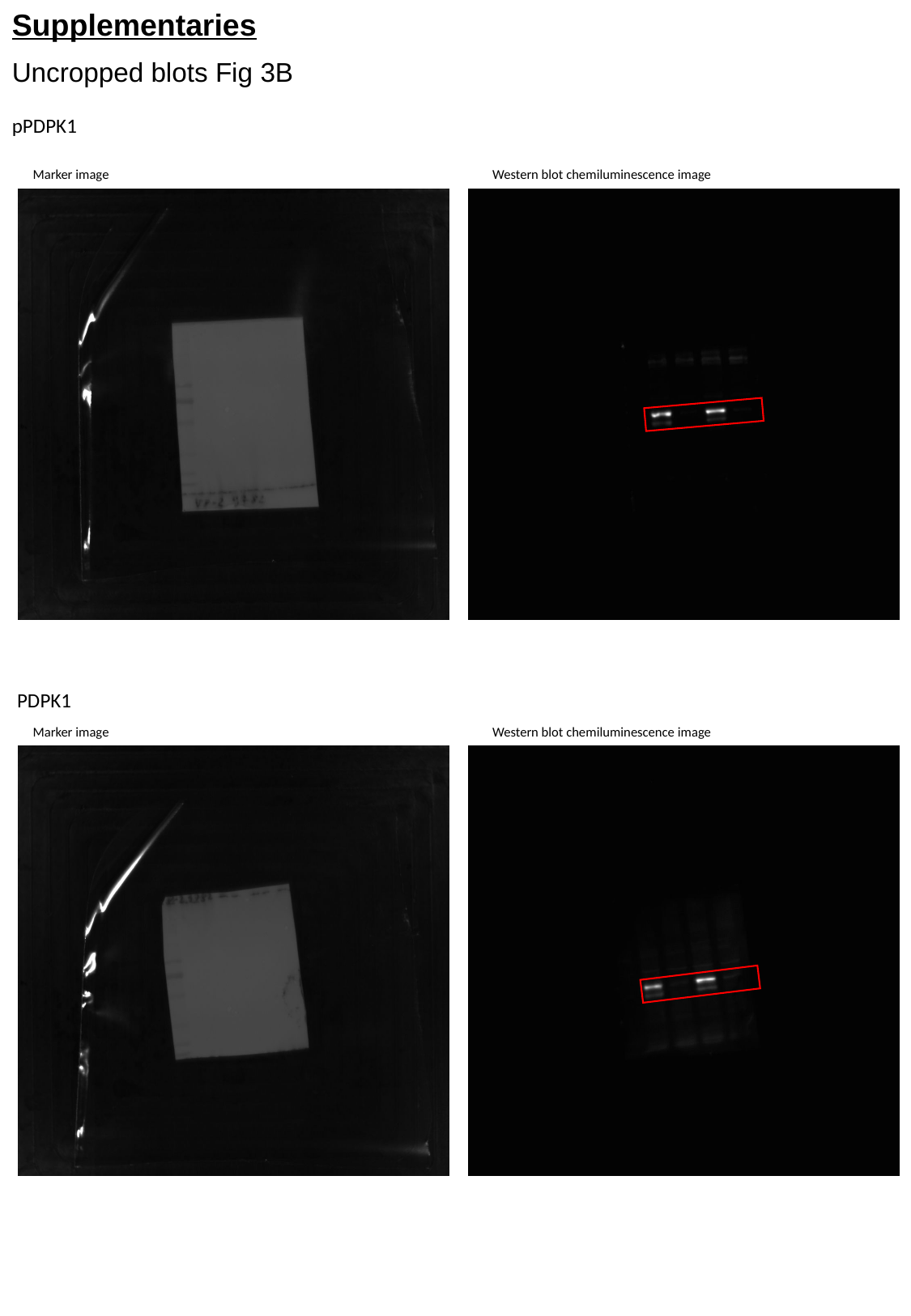

Supplementaries
Uncropped blots Fig 3B
pPDPK1
Marker image
Western blot chemiluminescence image
PDPK1
Marker image
Western blot chemiluminescence image

## Slide 10
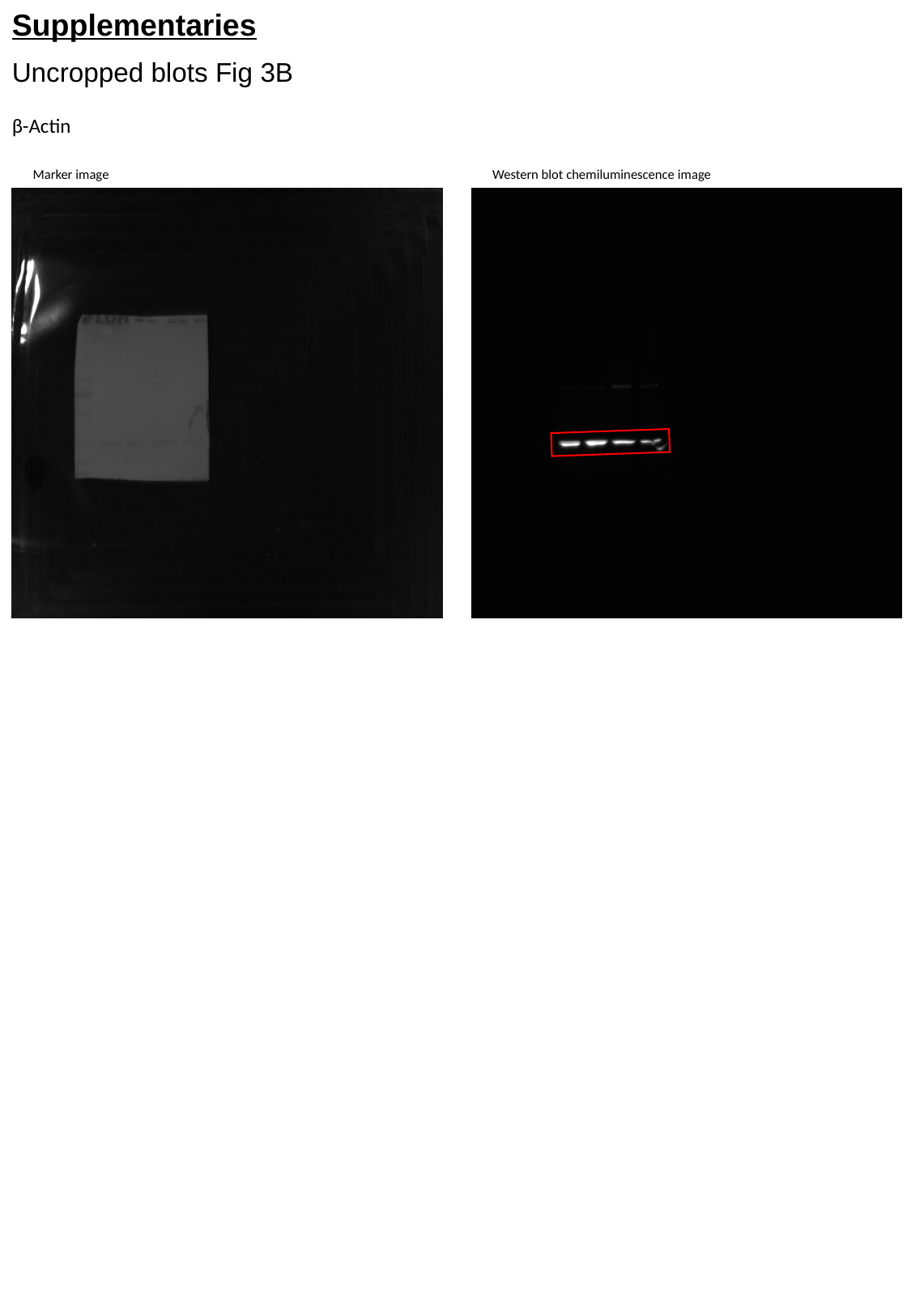

Supplementaries
Uncropped blots Fig 3B
β-Actin
Marker image
Western blot chemiluminescence image

## Slide 11
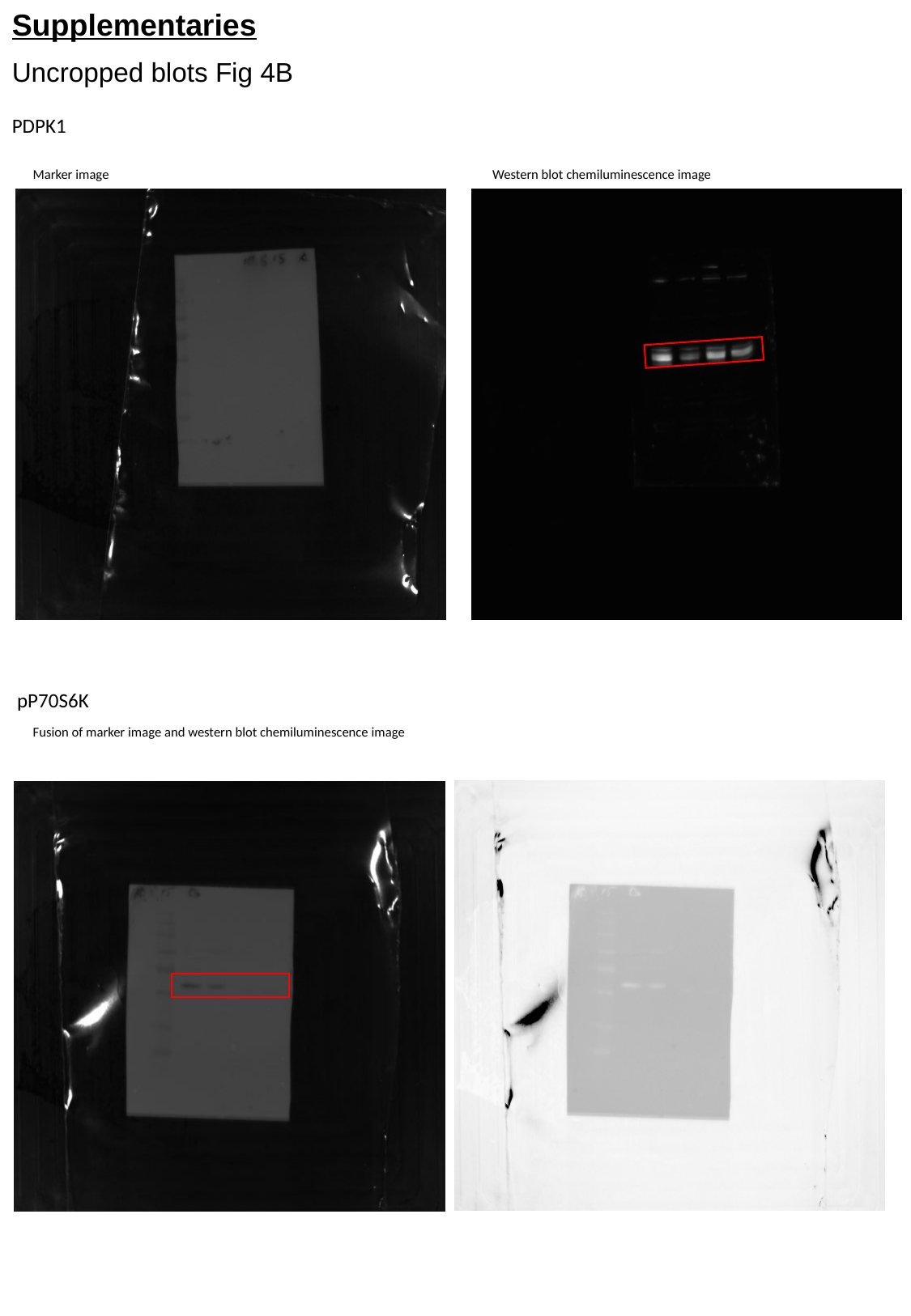

Supplementaries
Uncropped blots Fig 4B
PDPK1
Marker image
Western blot chemiluminescence image
pP70S6K
Fusion of marker image and western blot chemiluminescence image

## Slide 12
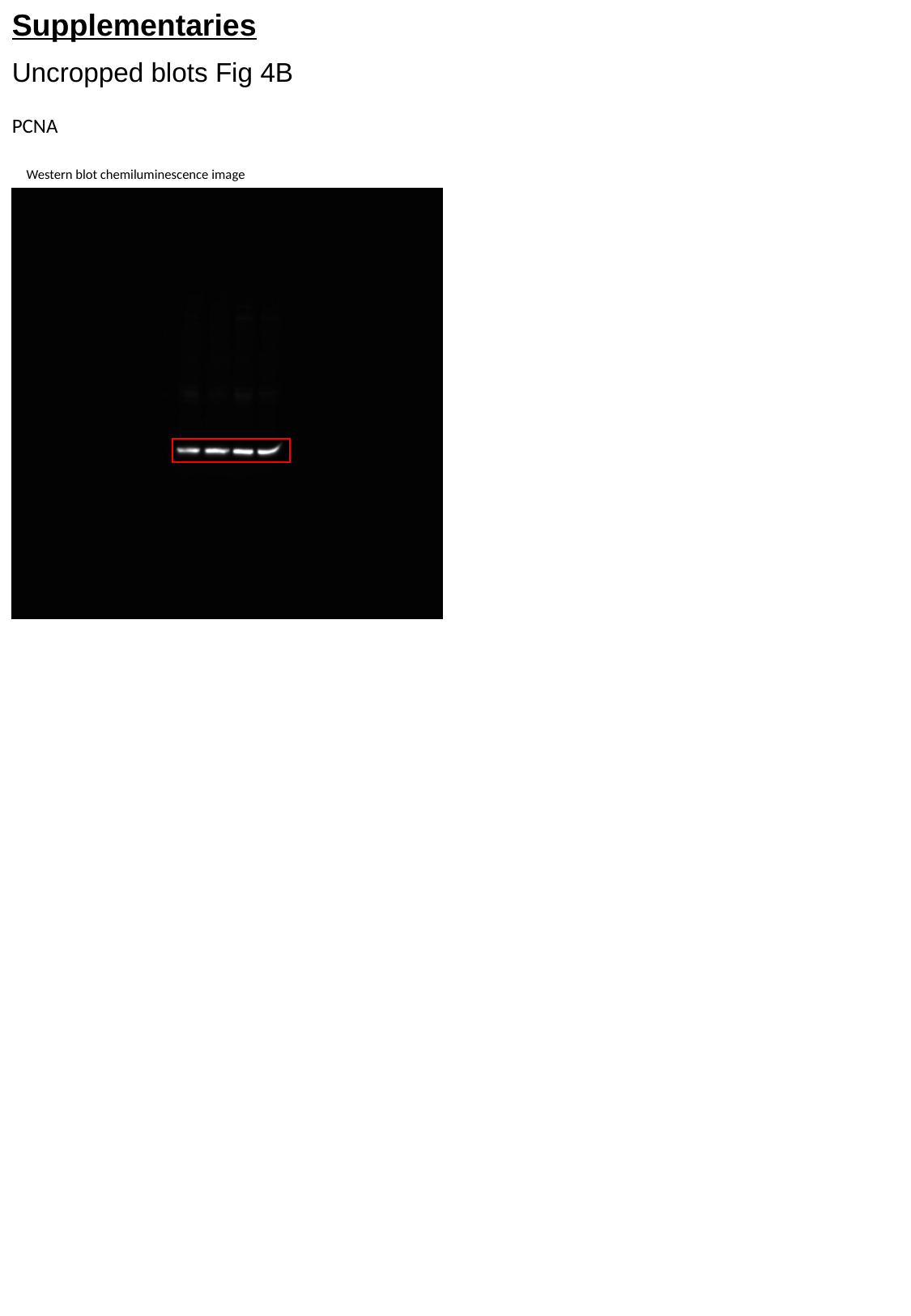

Supplementaries
Uncropped blots Fig 4B
PCNA
Western blot chemiluminescence image

## Slide 13
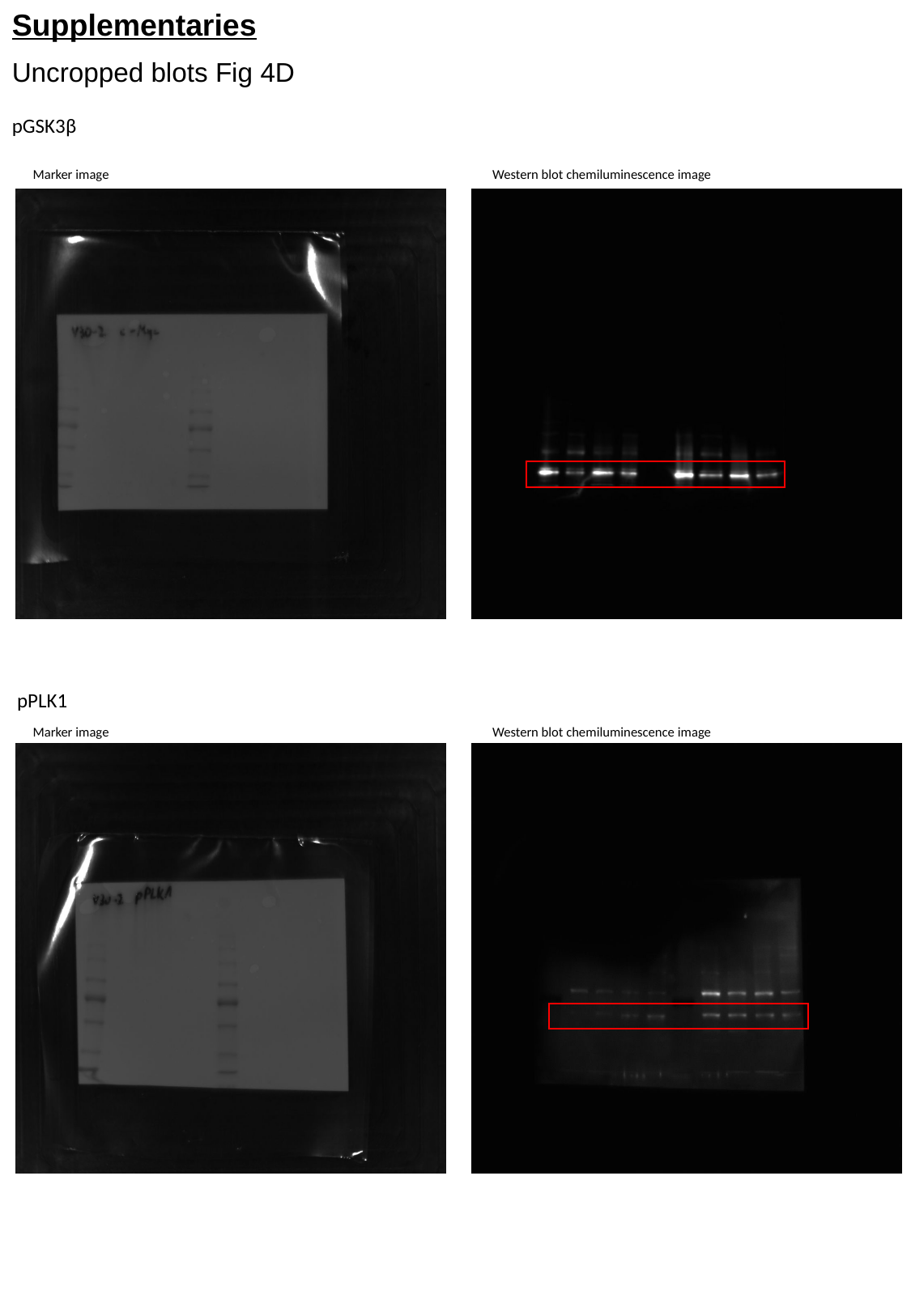

Supplementaries
Uncropped blots Fig 4D
pGSK3β
Marker image
Western blot chemiluminescence image
pPLK1
Marker image
Western blot chemiluminescence image

## Slide 14
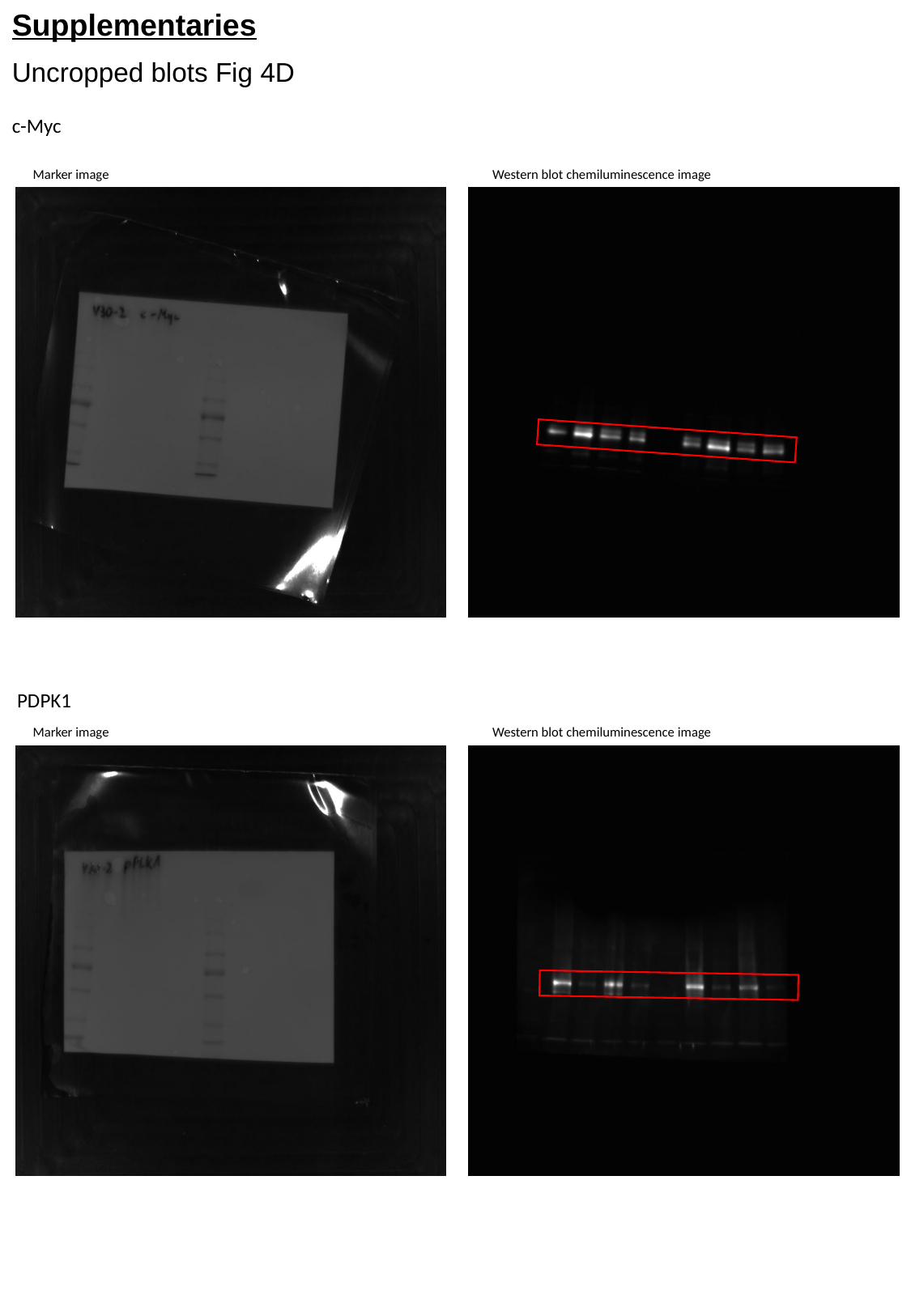

Supplementaries
Uncropped blots Fig 4D
c-Myc
Marker image
Western blot chemiluminescence image
PDPK1
Marker image
Western blot chemiluminescence image

## Slide 15
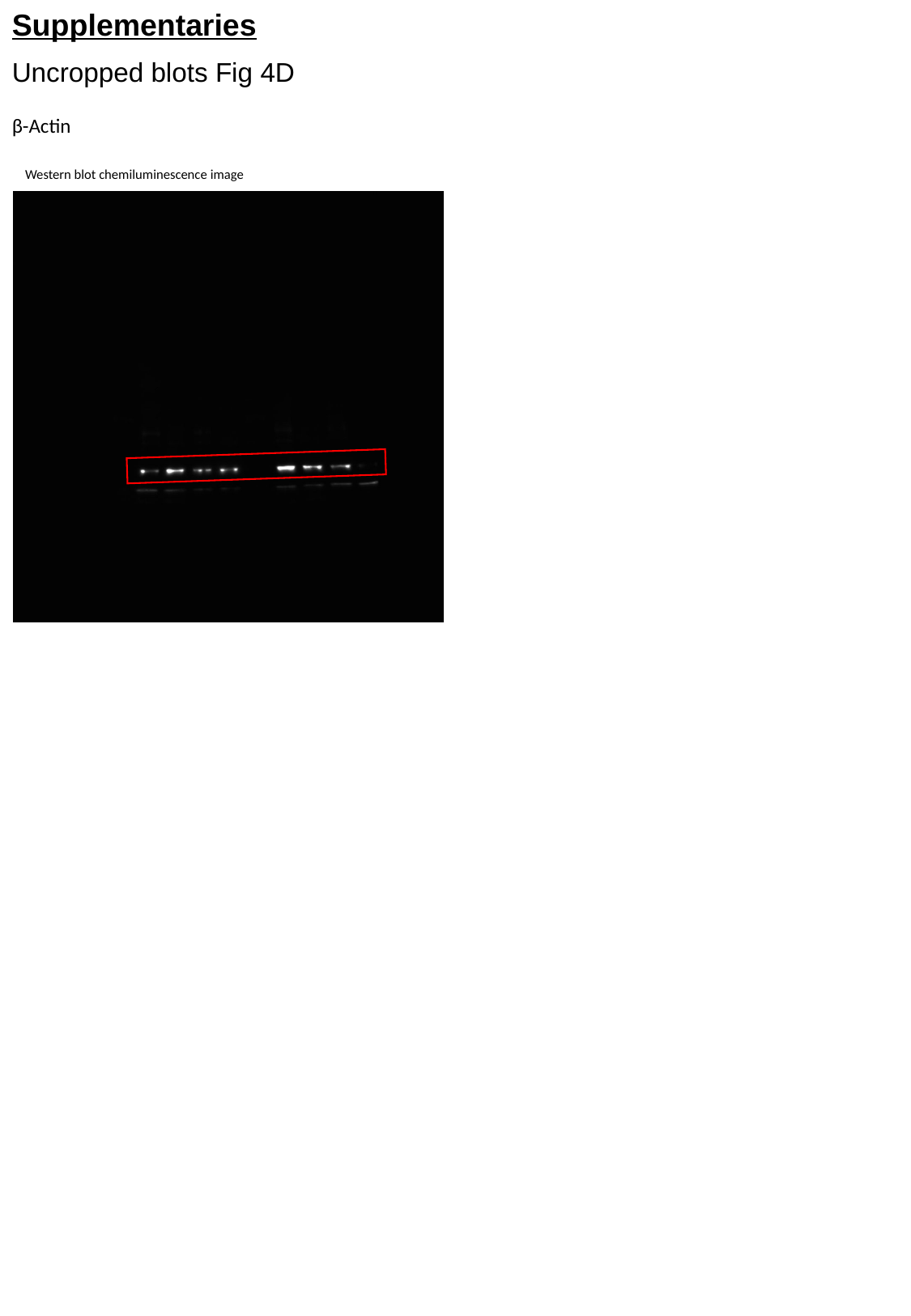

Supplementaries
Uncropped blots Fig 4D
β-Actin
Western blot chemiluminescence image

## Slide 16
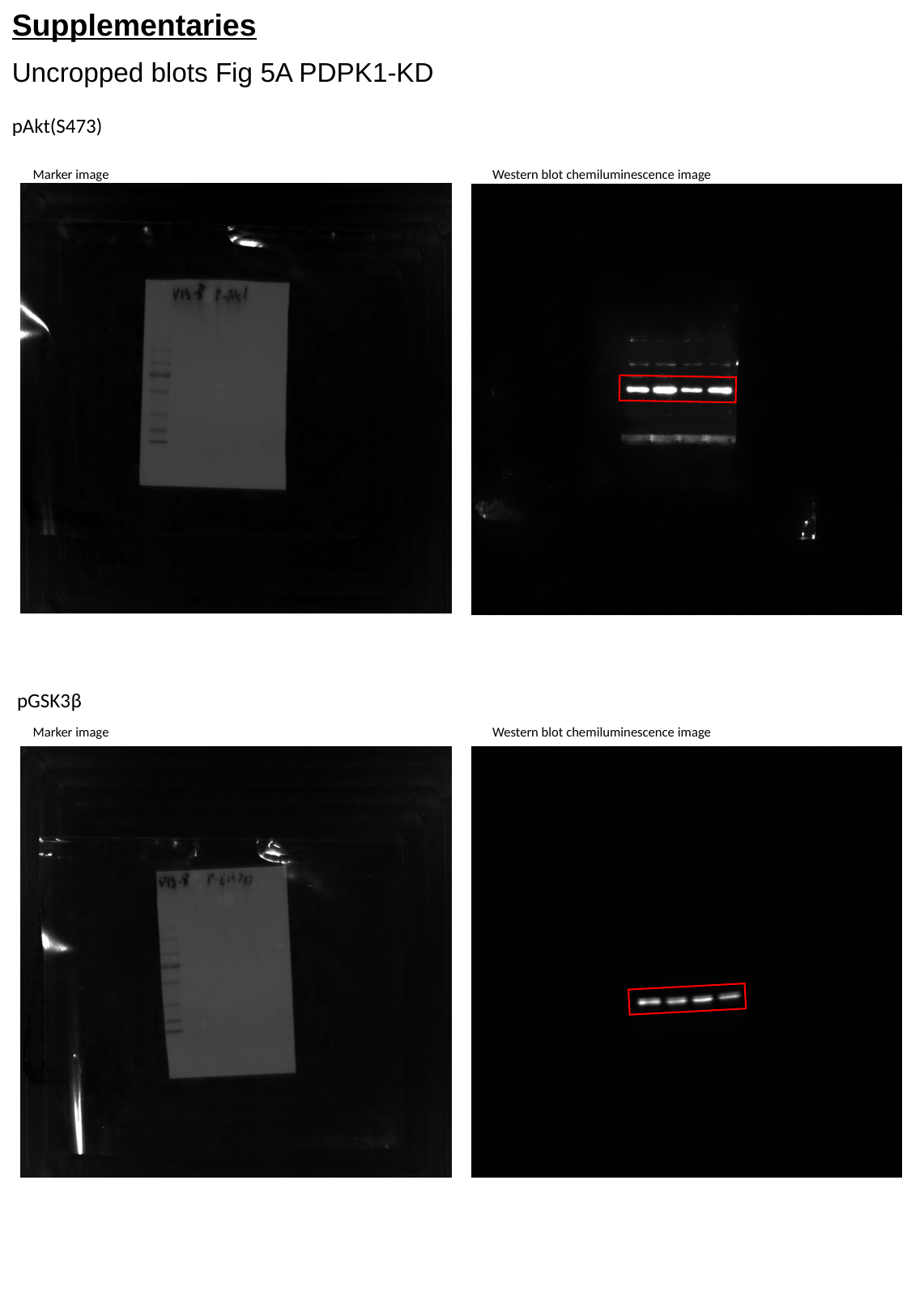

Supplementaries
Uncropped blots Fig 5A PDPK1-KD
pAkt(S473)
Marker image
Western blot chemiluminescence image
pGSK3β
Marker image
Western blot chemiluminescence image

## Slide 17
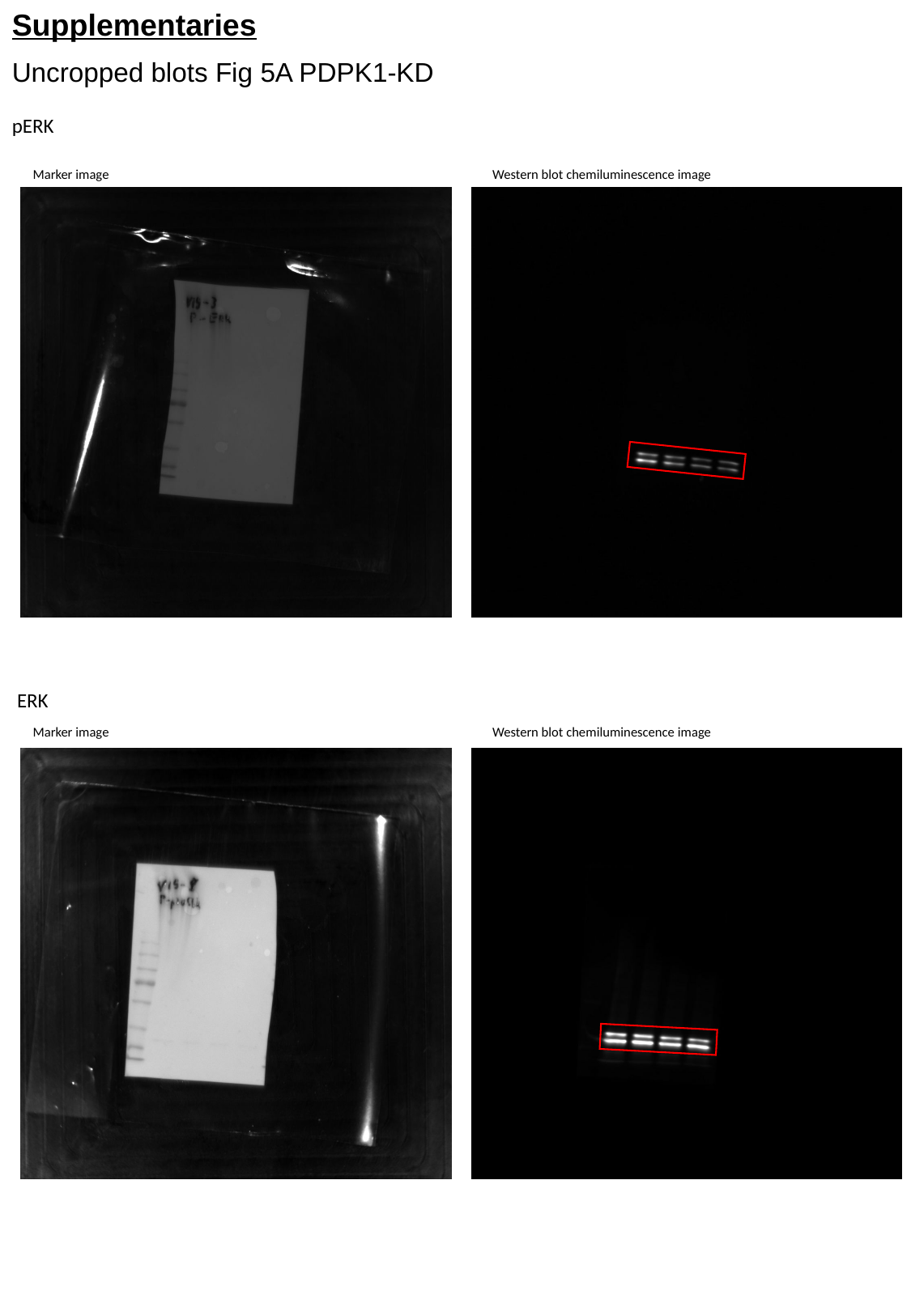

Supplementaries
Uncropped blots Fig 5A PDPK1-KD
pERK
Marker image
Western blot chemiluminescence image
ERK
Marker image
Western blot chemiluminescence image

## Slide 18
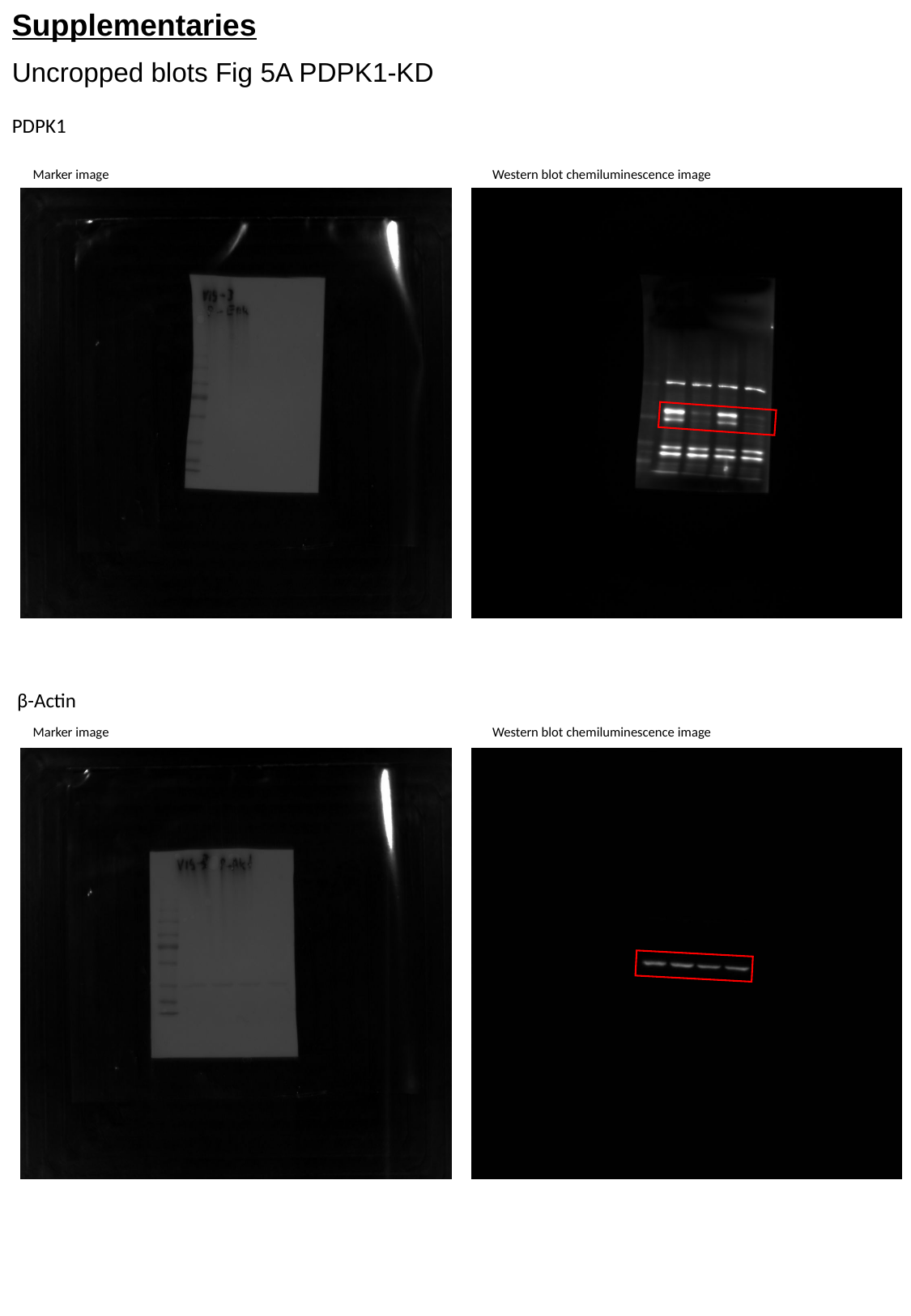

Supplementaries
Uncropped blots Fig 5A PDPK1-KD
PDPK1
Marker image
Western blot chemiluminescence image
β-Actin
Marker image
Western blot chemiluminescence image

## Slide 19
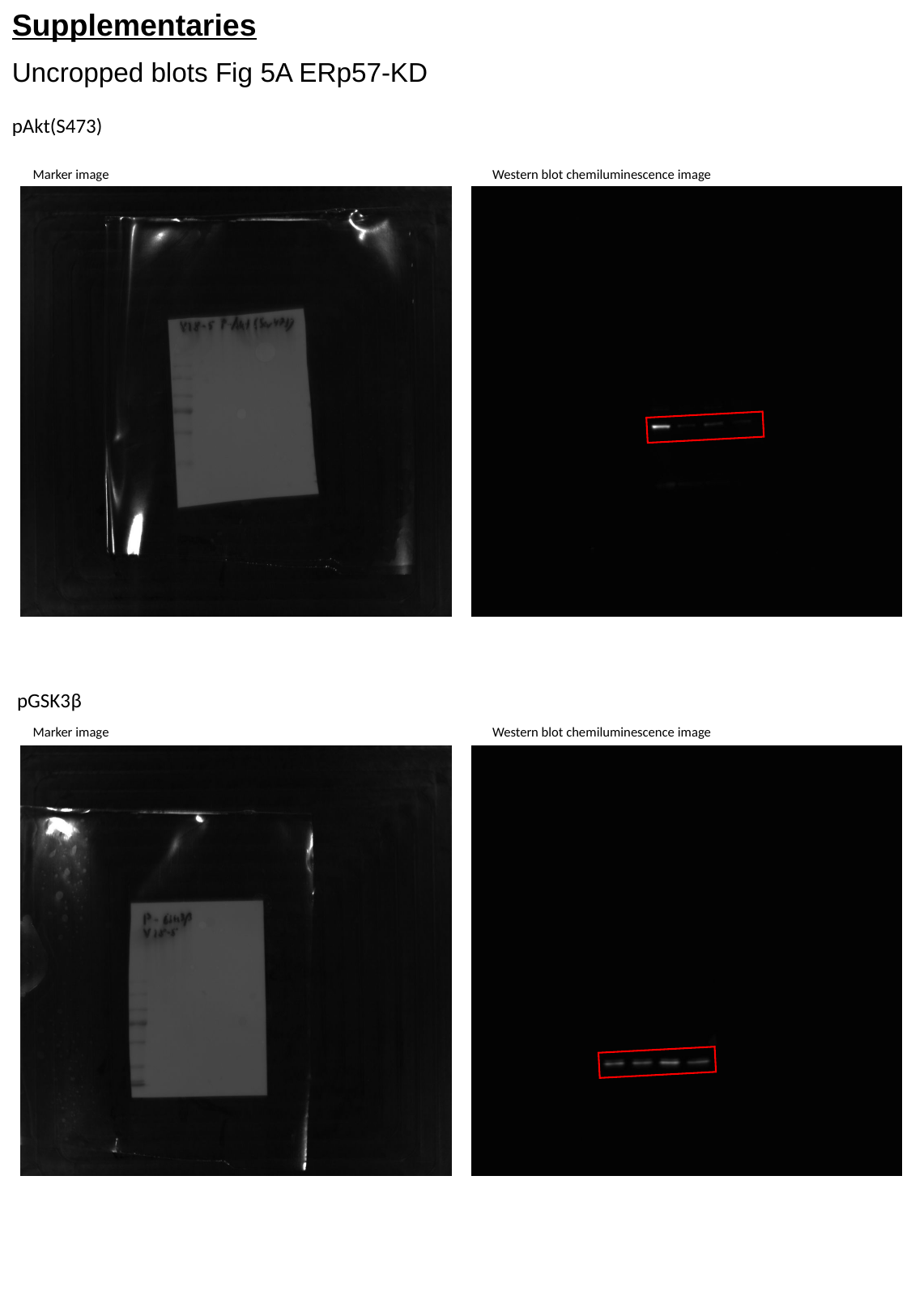

Supplementaries
Uncropped blots Fig 5A ERp57-KD
pAkt(S473)
Marker image
Western blot chemiluminescence image
pGSK3β
Marker image
Western blot chemiluminescence image

## Slide 20
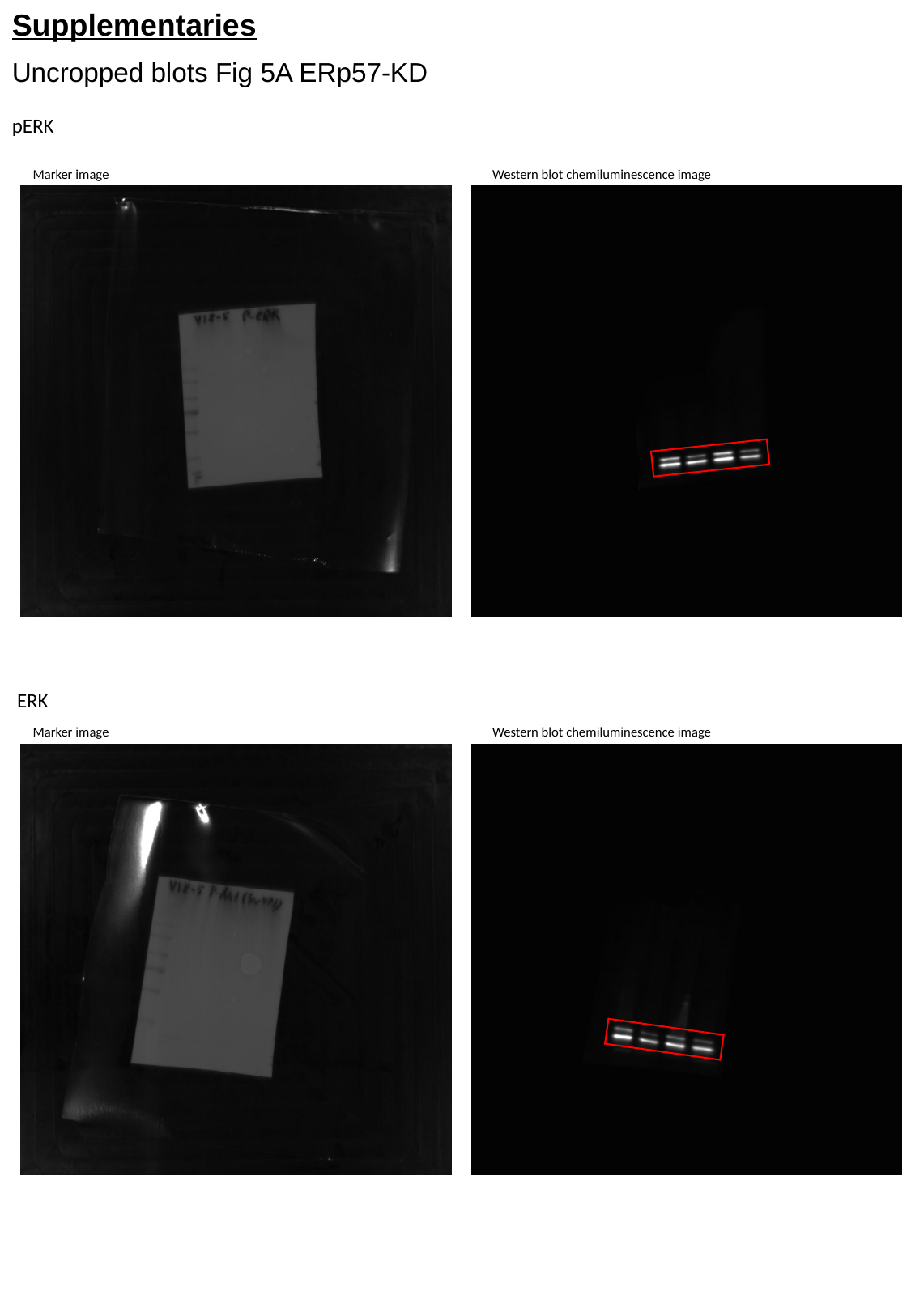

Supplementaries
Uncropped blots Fig 5A ERp57-KD
pERK
Marker image
Western blot chemiluminescence image
ERK
Marker image
Western blot chemiluminescence image

## Slide 21
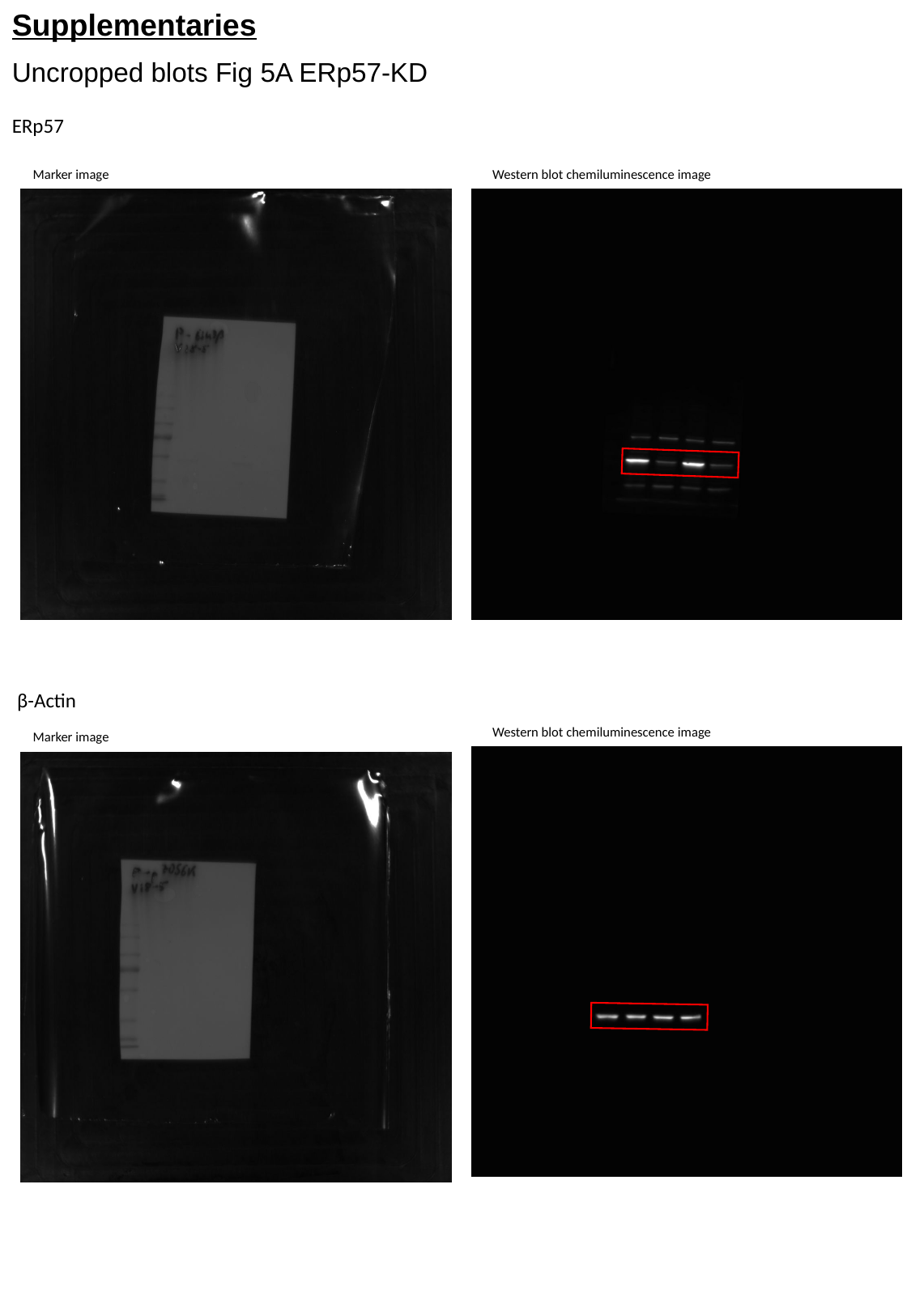

Supplementaries
Uncropped blots Fig 5A ERp57-KD
ERp57
Marker image
Western blot chemiluminescence image
β-Actin
Western blot chemiluminescence image
Marker image

## Slide 22
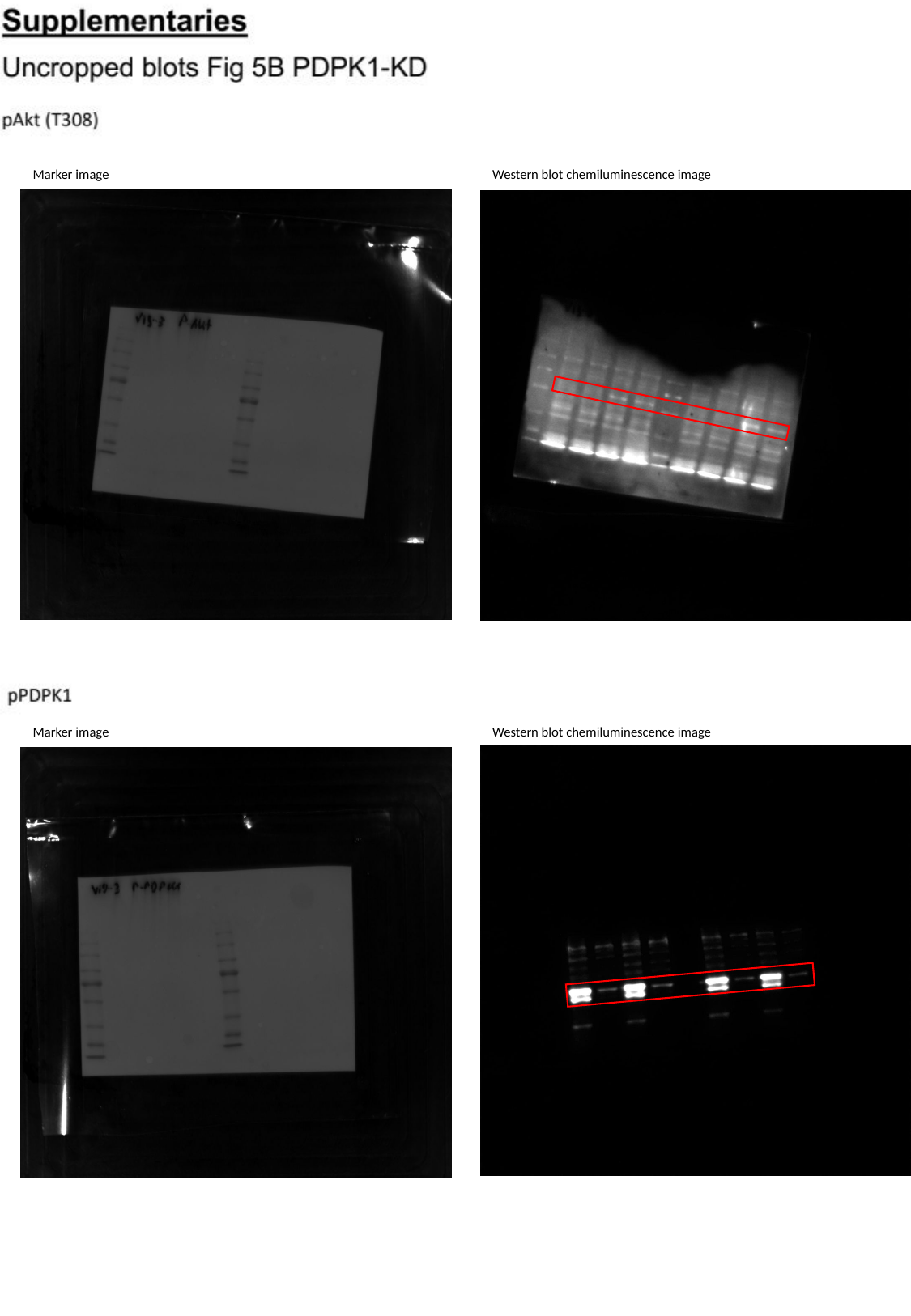

Supplementaries
Uncropped blots Fig 5B PDPK1-KD
pAkt (T308)
Marker image
Western blot chemiluminescence image
pPDPK1
Marker image
Western blot chemiluminescence image

## Slide 23
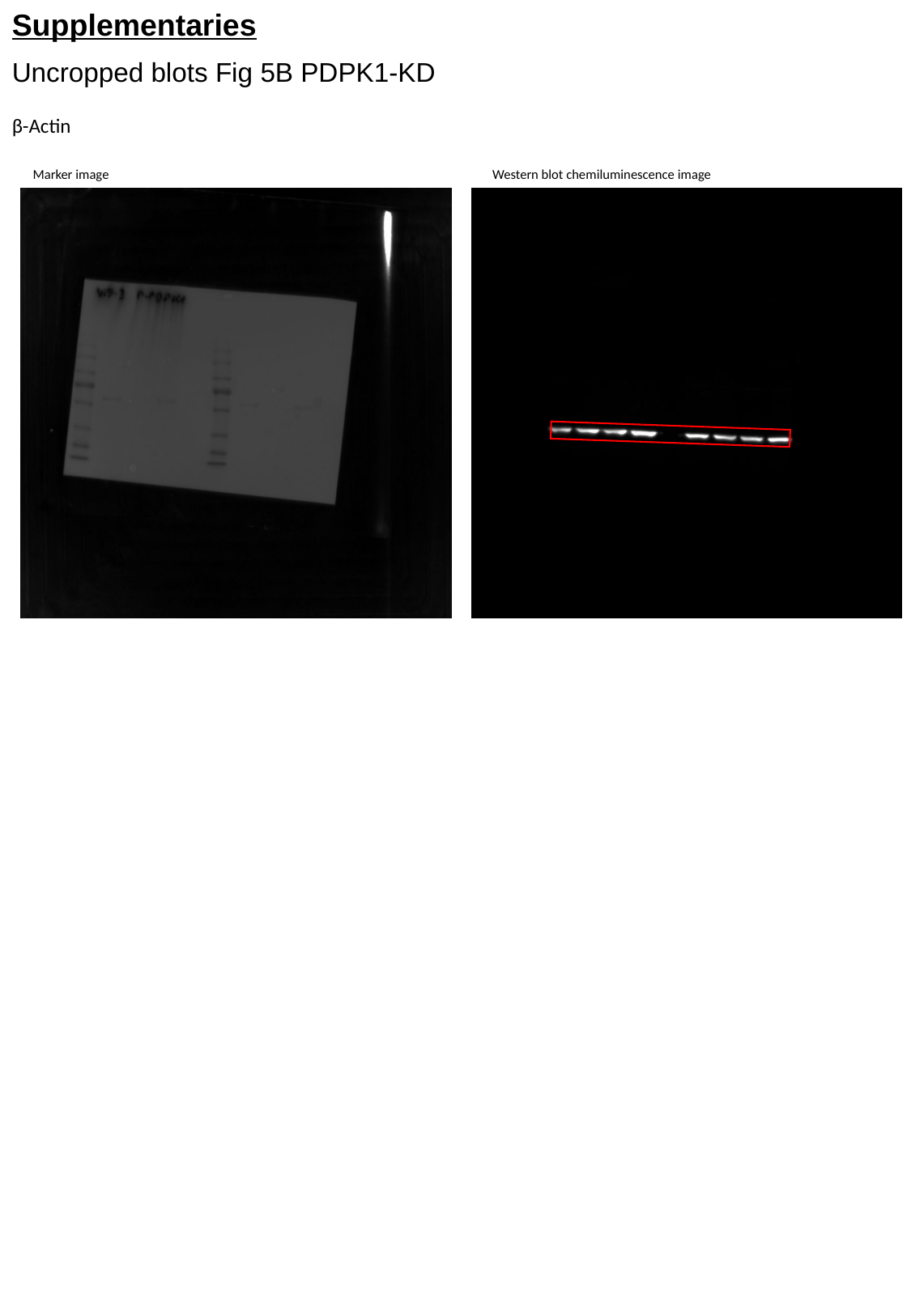

Supplementaries
Uncropped blots Fig 5B PDPK1-KD
β-Actin
Marker image
Western blot chemiluminescence image

## Slide 24
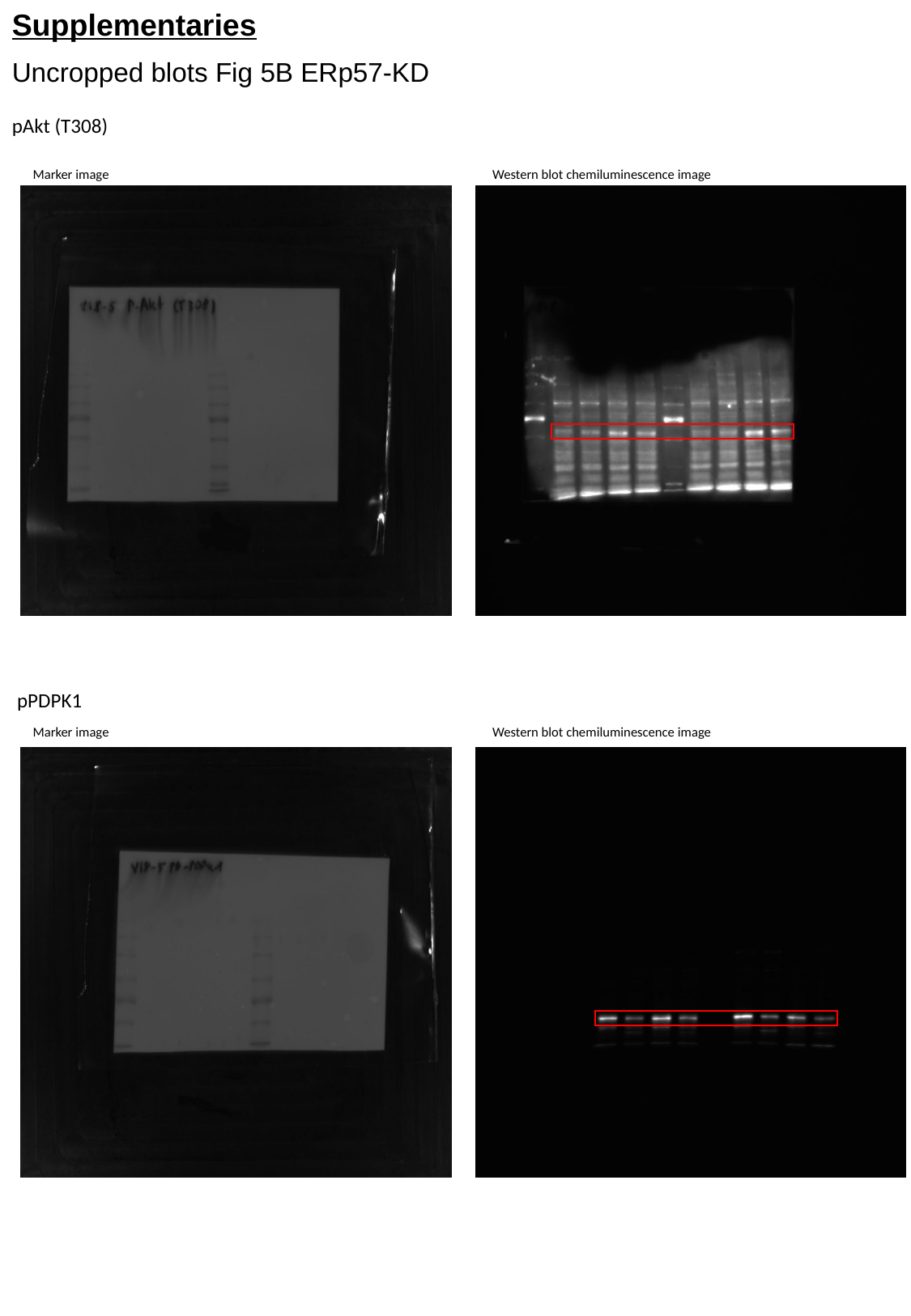

Supplementaries
Uncropped blots Fig 5B ERp57-KD
pAkt (T308)
Marker image
Western blot chemiluminescence image
pPDPK1
Marker image
Western blot chemiluminescence image

## Slide 25
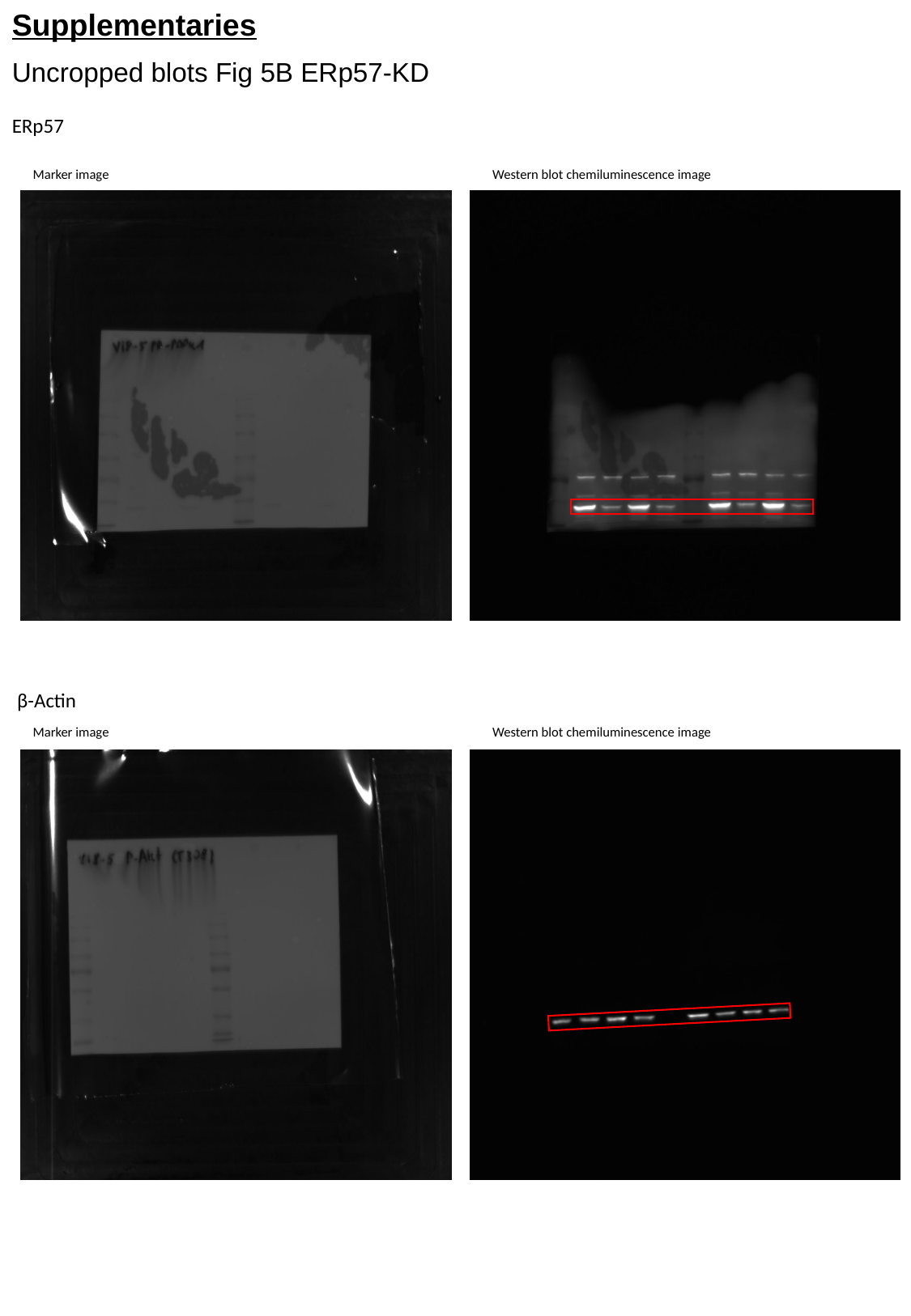

Supplementaries
Uncropped blots Fig 5B ERp57-KD
ERp57
Marker image
Western blot chemiluminescence image
β-Actin
Marker image
Western blot chemiluminescence image

## Slide 26
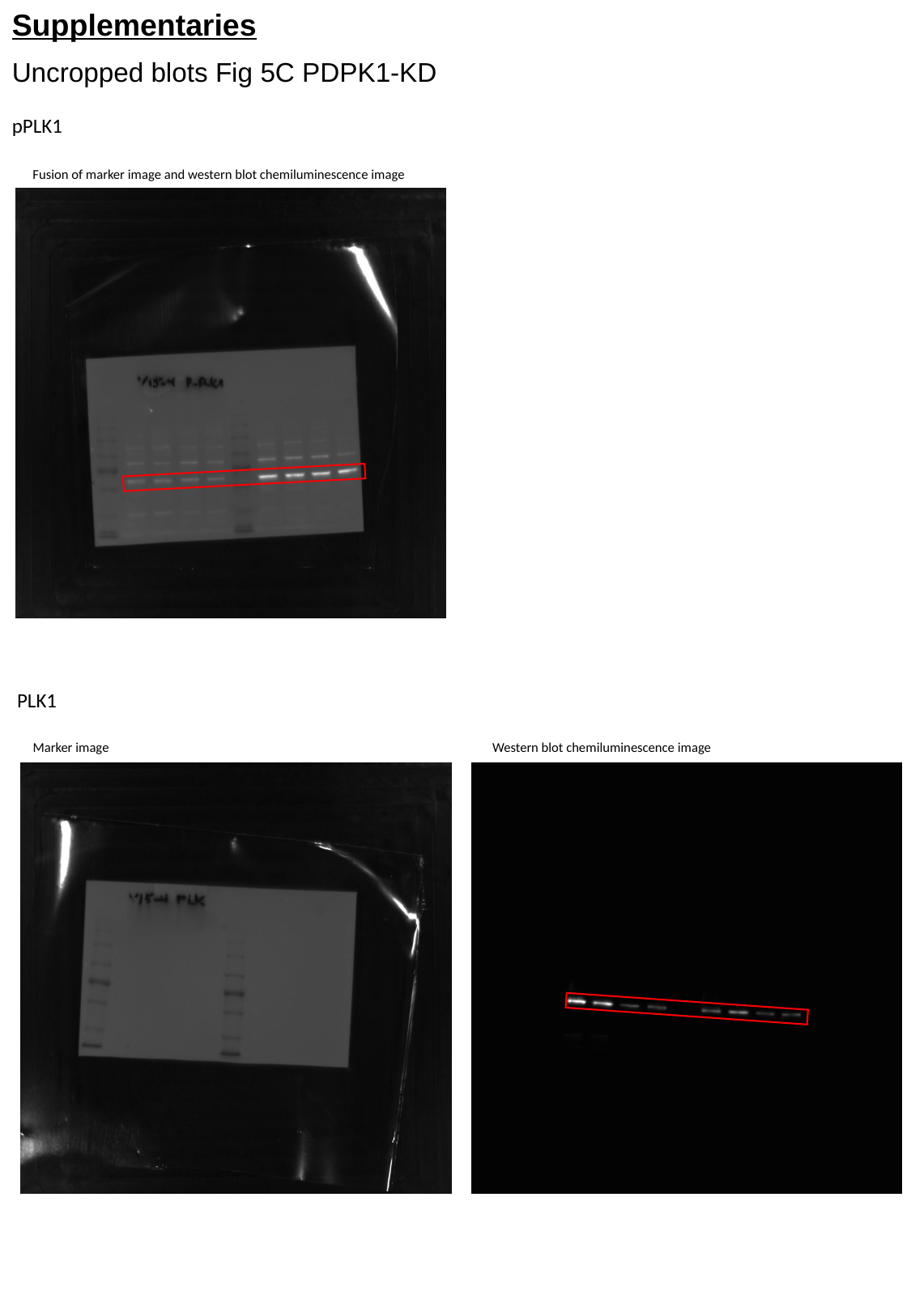

Supplementaries
Uncropped blots Fig 5C PDPK1-KD
pPLK1
Fusion of marker image and western blot chemiluminescence image
PLK1
Marker image
Western blot chemiluminescence image

## Slide 27
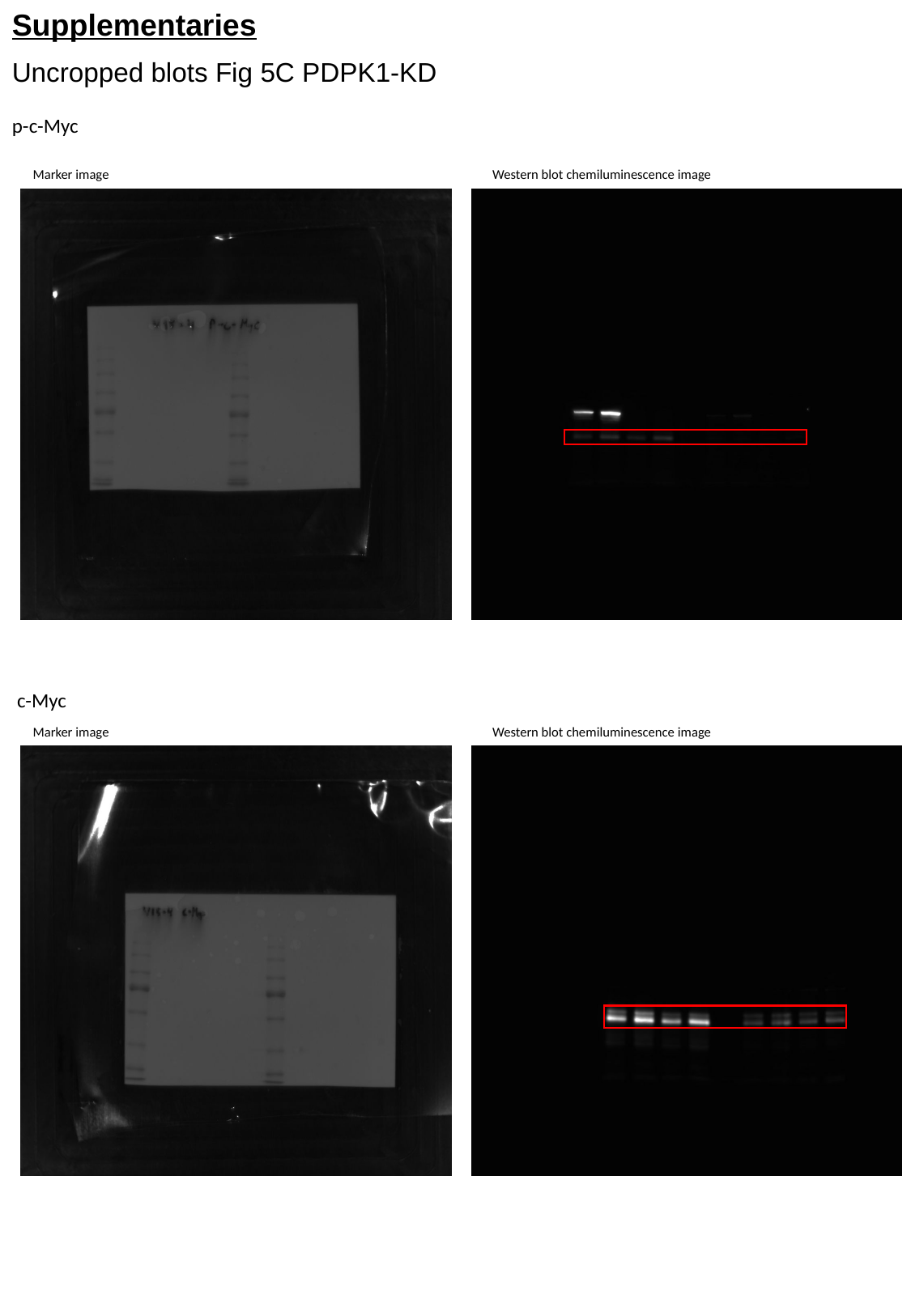

Supplementaries
Uncropped blots Fig 5C PDPK1-KD
p-c-Myc
Marker image
Western blot chemiluminescence image
c-Myc
Marker image
Western blot chemiluminescence image

## Slide 28
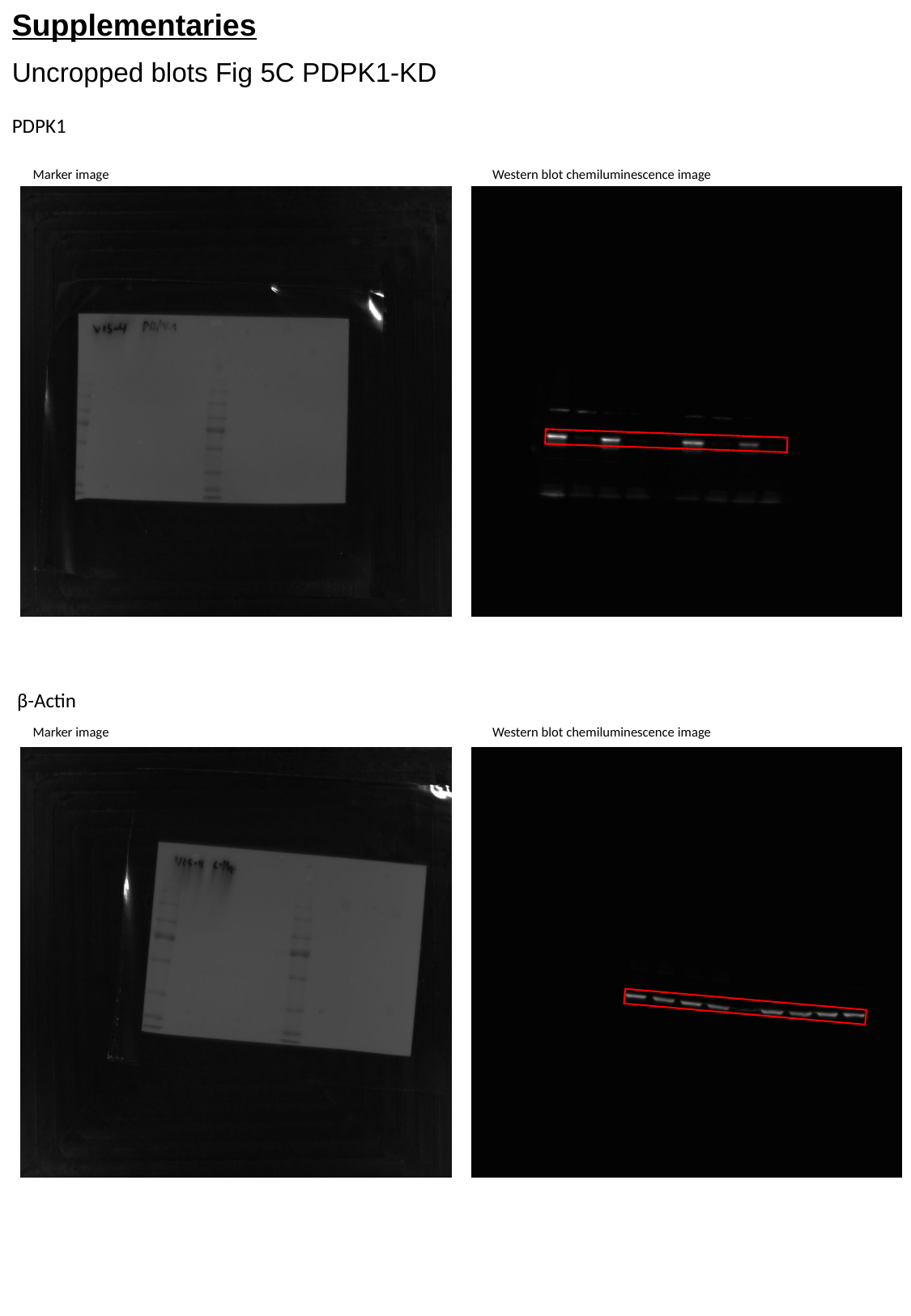

Supplementaries
Uncropped blots Fig 5C PDPK1-KD
PDPK1
Marker image
Western blot chemiluminescence image
β-Actin
Marker image
Western blot chemiluminescence image

## Slide 29
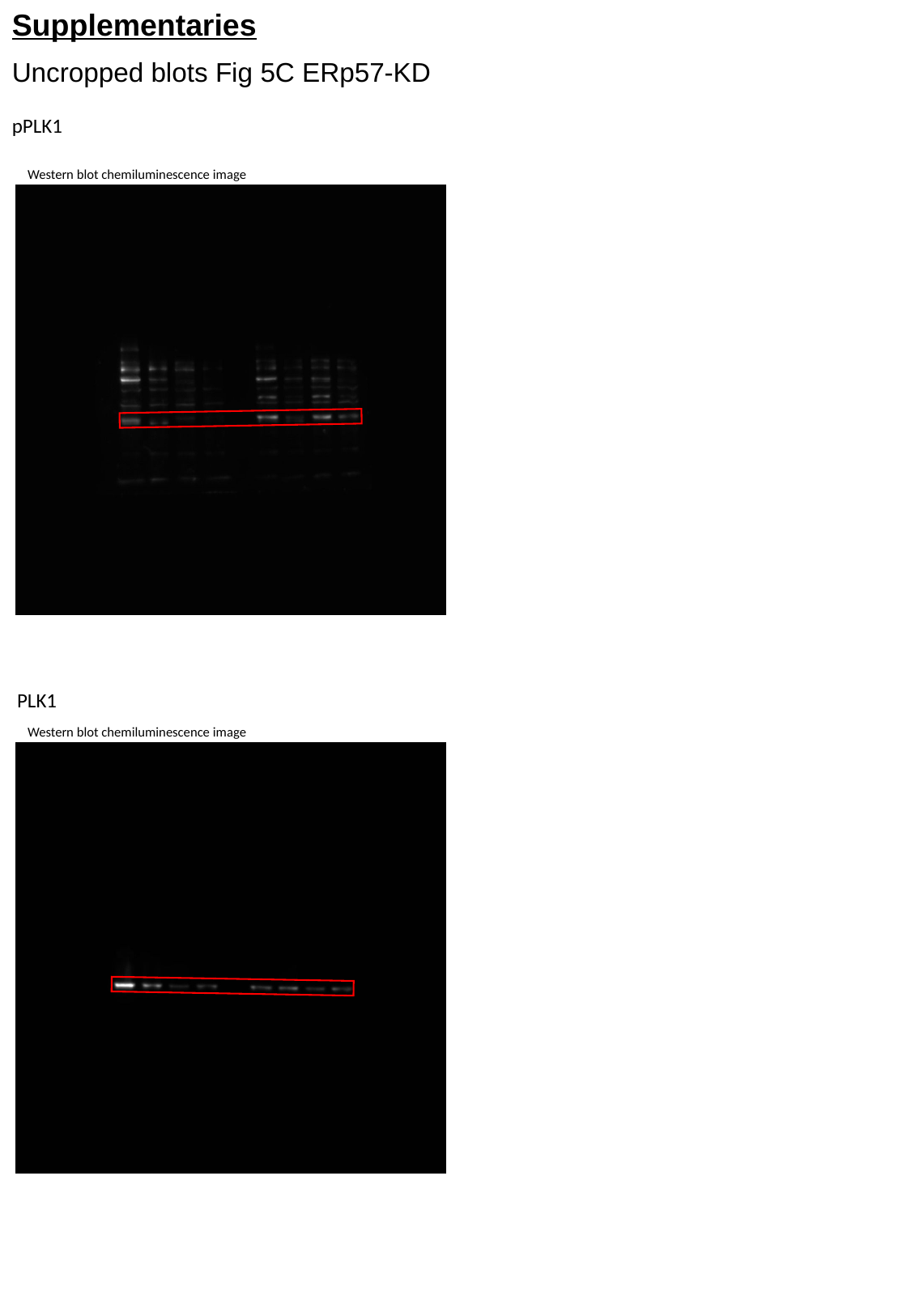

Supplementaries
Uncropped blots Fig 5C ERp57-KD
pPLK1
Western blot chemiluminescence image
PLK1
Western blot chemiluminescence image

## Slide 30
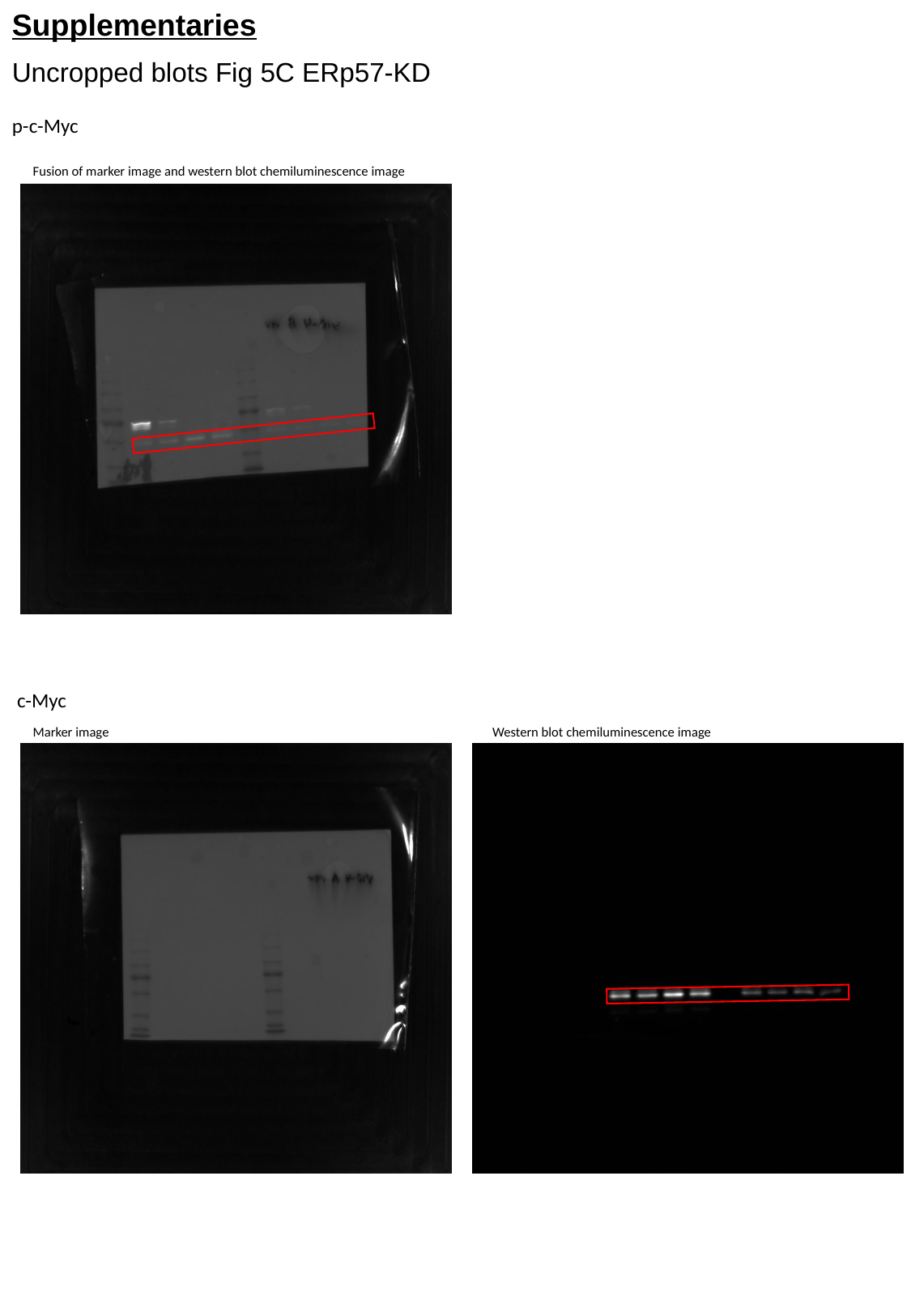

Supplementaries
Uncropped blots Fig 5C ERp57-KD
p-c-Myc
Fusion of marker image and western blot chemiluminescence image
c-Myc
Marker image
Western blot chemiluminescence image

## Slide 31
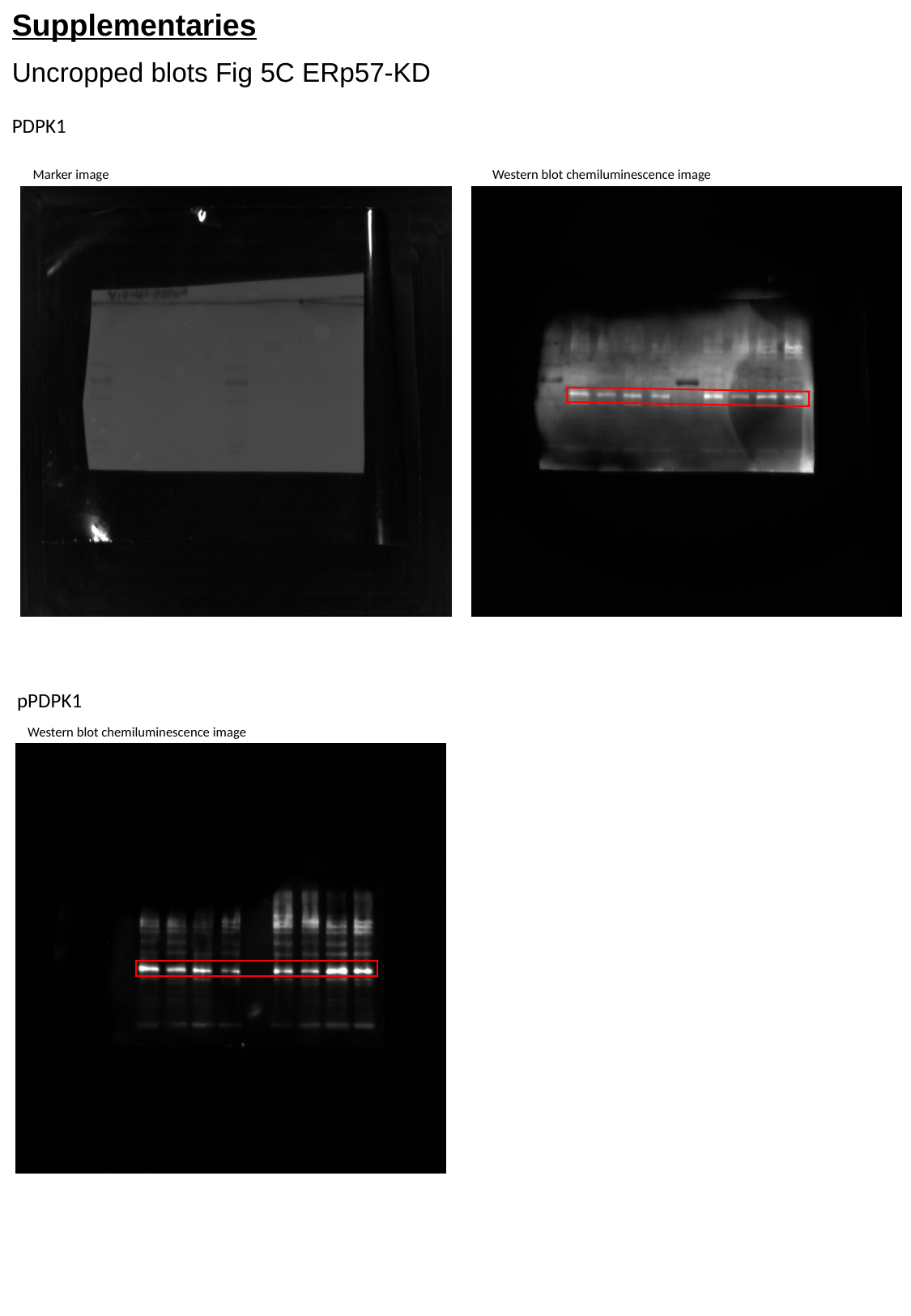

Supplementaries
Uncropped blots Fig 5C ERp57-KD
PDPK1
Marker image
Western blot chemiluminescence image
pPDPK1
Western blot chemiluminescence image

## Slide 32
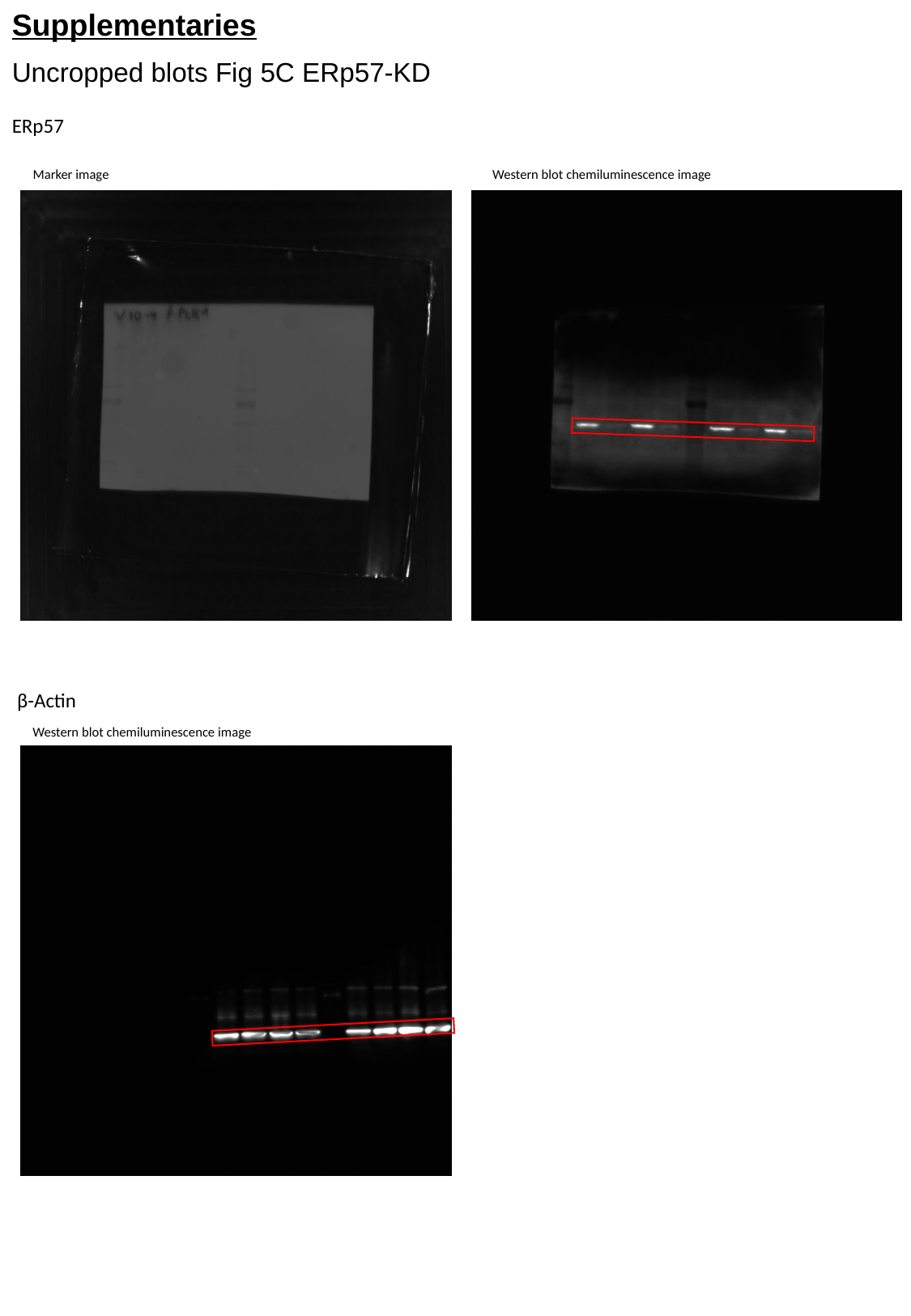

Supplementaries
Uncropped blots Fig 5C ERp57-KD
ERp57
Marker image
Western blot chemiluminescence image
β-Actin
Western blot chemiluminescence image

## Slide 33
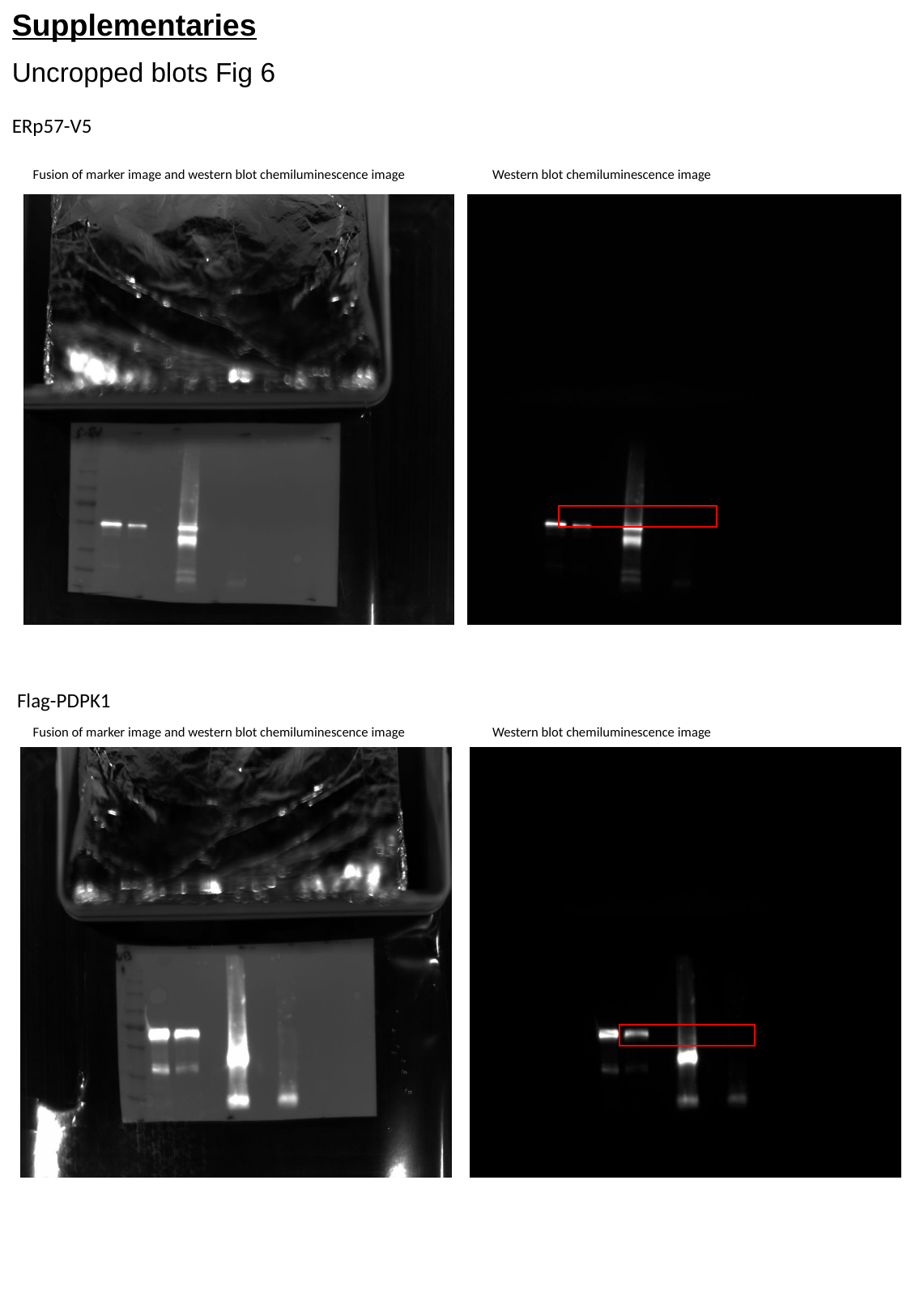

Supplementaries
Uncropped blots Fig 6
ERp57-V5
Fusion of marker image and western blot chemiluminescence image
Western blot chemiluminescence image
Flag-PDPK1
Fusion of marker image and western blot chemiluminescence image
Western blot chemiluminescence image

## Slide 34
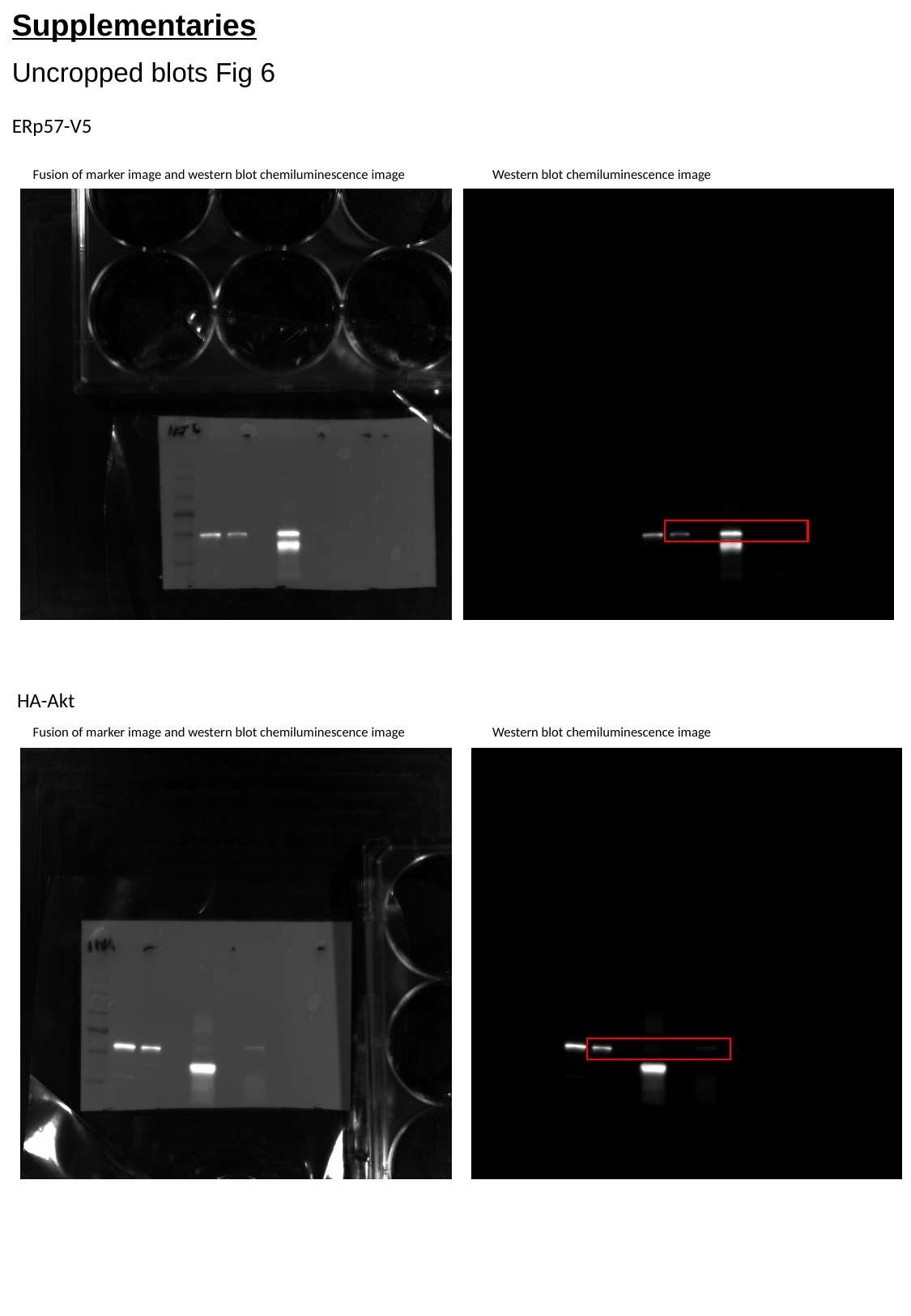

Supplementaries
Uncropped blots Fig 6
ERp57-V5
Fusion of marker image and western blot chemiluminescence image
Western blot chemiluminescence image
HA-Akt
Fusion of marker image and western blot chemiluminescence image
Western blot chemiluminescence image

## Slide 35
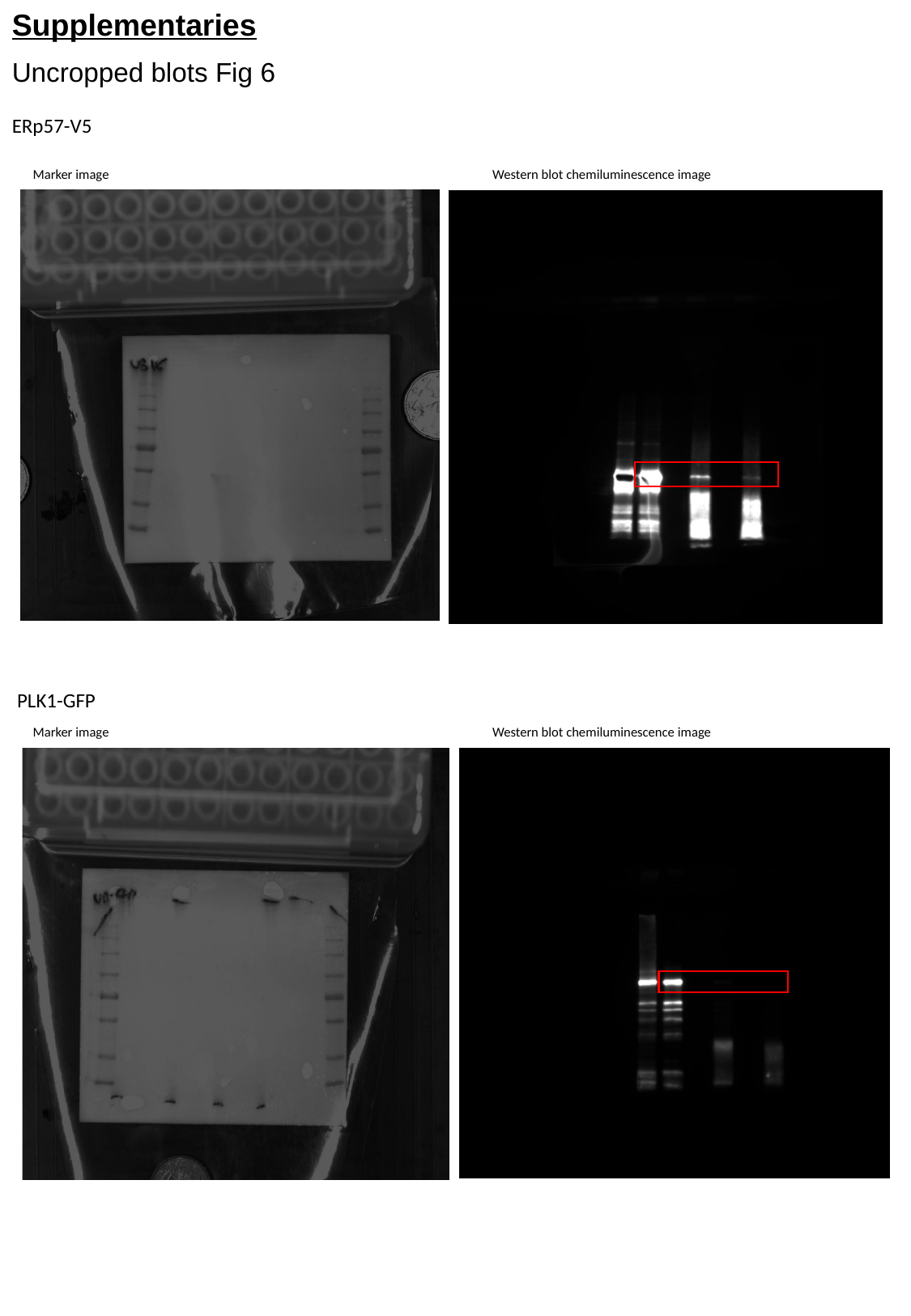

Supplementaries
Uncropped blots Fig 6
ERp57-V5
Marker image
Western blot chemiluminescence image
PLK1-GFP
Marker image
Western blot chemiluminescence image

## Slide 36
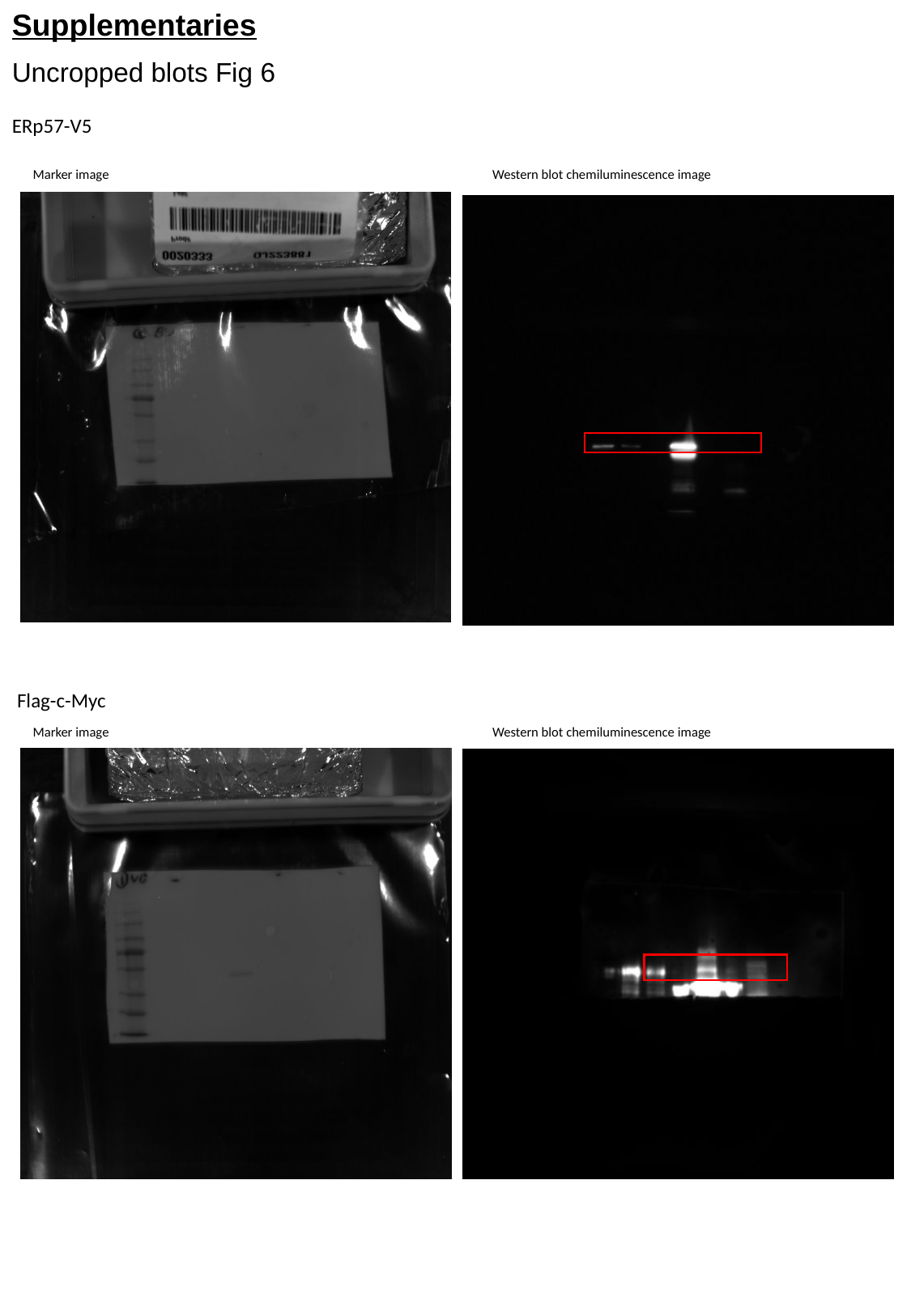

Supplementaries
Uncropped blots Fig 6
ERp57-V5
Marker image
Western blot chemiluminescence image
Flag-c-Myc
Marker image
Western blot chemiluminescence image
